# Proteomic profiling of the *Pseudomonas aeruginosa* stringent response highlights global adaptive responses during stationary phase

**DOI:** 10.1128/msystems.01742-25

**Published:** 2026-09-17

**Authors:** Jacquelyn Rich, Samuel J. T. Wardell, Dorival Martins, Malika Khakhimova, Gowthami Sampathkumar, Tevy Chan, Thi Tuyet Mai Nguyen, Geoffrey McKay, Lorne Taylor, Daniel Pletzer, Dao Nguyen

**Affiliations:** 1 Department of Microbiology and Immunology, McGill University 5620, Montreal, Canada; 2 Department of Microbiology and Immunology, Faculty of Biomedical Sciences, University of Otago 2495, Dunedin, New Zealand; 3 Division of Geriatric Medicine, Department of Medicine, McGill University 246536, Montreal, QC, Canada; 4 Department of Medicine, McGill University 5620, Montreal, Canada; 5 Meakins-Christie Laboratories, Research Institute of the McGill University Health Centre 507266, Montreal, Canada; 6 Proteomics and Molecular Analysis Platform, Research Institute of the McGill University Health Centre 507266, Montreal, Canada; University of Minnesota Twin Cities, Minneapolis, Minnesota, USA

**Keywords:** *Pseudomonas aeruginosa*, stringent response, stationary phase, proteomics, transcriptomics, stress adaptation

## Abstract

*Pseudomonas aeruginosa* (*P.a*) is a clinically important opportunistic pathogen capable of adapting and surviving a wide range of environments and stress conditions. A central regulator of this adaptability is the stringent response, mediated by the alarmones (p)ppGpp. Activation of the stringent response triggers a global physiological shift away from active metabolism toward stress management and cellular quiescence, a key transition to stationary phase. To date, few studies have investigated the protein-level processes under stringent response control. In this study, we performed shotgun proteomic profiling (LC-MS/MS) of a (p)ppGpp-null *relA spoT* double knockout mutant (ΔSR) and its isogenic PAO1 parent during late exponential and stationary phase planktonic growth in rich medium. In this first characterization of the stringent response proteome in *P.a*, we identified 146 differentially abundant proteins (DAP) in late exponential phase ΔSR cells, and 2.3-fold greater DAP (339) in stationary-phase cells. Functional characterization and system-level analysis of the differentially abundant stationary phase proteome highlighted the key role of (p)ppGpp in shutting down a wide range of metabolic processes while inducing adaptive processes such as responses to oxidative stress. Comparative RNA-seq analysis also uncovered incongruent protein and mRNA responses in the ΔSR relative to wild-type cells in several processes, including iron homeostasis and antioxidant defenses. Transcriptomic analysis of the ΔSR also revealed differential expression of numerous sRNAs, including PrrF1/2, raising the possibility that sRNAs may mediate certain stringent-response-dependent post-transcriptional control. Phenotypic validation of the ΔSR proteome confirmed multiple defects in antioxidant defenses and dysregulated pyoverdine biosynthesis.

**IMPORTANCE:**

*Pseudomonas aeruginosa* is an important opportunistic human pathogen that causes difficult-to-treat infections and is highly adaptable to stressful environments. A key orchestrator of this adaptability is the stringent response, mediated by the signaling molecule (p)ppGpp. In this study, we characterized the extensive proteome remodeling in the (p)ppGpp-null *relA spoT* mutant during late exponential and stationary phase and provided the first protein-level analysis of the stringent response in *P. aeruginosa*. These data highlight the role of (p)ppGpp in shutting down broad metabolic functions and activating adaptive pathways, including stress-resistance responses, particularly during stationary phase. Using comparative proteomics and RNA-seq, we also find that several processes—such as iron regulation and oxidative stress responses—show mismatched RNA and protein changes, suggesting additional layers of post-transcriptional control, potentially involving small regulatory RNAs.

## INTRODUCTION

The ability to sense and adapt to environmental fluctuations and stressors is critical for the long-term survival of bacteria. *Pseudomonas aeruginosa* (*P.a*), renowned for its metabolic versatility, thrives in a wide range of natural environments and clinical settings where it is a formidable opportunistic human pathogen (1, 2). *P.a* causes both acute and chronic infections, most notably recalcitrant infections commonly observed in cystic fibrosis lung disease (3, 4). In natural and host environments, *P.a* encounters stressors, including nutrient limitation, oxidative stress, and antimicrobials, and must adapt to mitigate cell damage or death.

The stringent response is a widely conserved pleiotropic response to nutrient and environmental stress. This response is mediated by the accumulation of the signaling molecules ppGpp and pppGpp, known collectively as the alarmone (p)ppGpp (5), and by the co-regulator DksA, which binds RNA polymerase to potentiate transcription during the stringent response (6, 7). In most *Gammaproteobacteria*, including *Pa*, (p)ppGpp is synthesized by RelA*,* a monofunctional synthetase, and SpoT, a bifunctional synthetase with (p)ppGpp hydrolase activity (8). *P.a* mutants lacking both RelA and SpoT do not produce (p)ppGpp (9–11).

Although (p)ppGpp contributes to basal cellular physiology and modulates the expression of thousands of genes even under optimal growth conditions (11), its concentration rises upon nutrient depletion (e.g., amino acid, carbon, fatty acid, or iron), various stressors (e.g., pH, osmotic), and transition to stationary phase (7, 12, 13). In *Escherichia coli*, as a model for *Gammaproteobacteria*, accumulation of (p)ppGpp induces major shifts in gene transcription. This results from direct binding to the RNA polymerase and indirect effects through the enhanced expression and activity of alternative sigma factors, notably the stationary phase factor RpoS, stress-responsive sigma factors RpoE and RpoH (14–17). Downstream altered expression of transcriptional regulators, such as *lasR*, *rhlR*, and *algR* in *P.a* (11, 14, 15), exert further indirect effects. (p)ppGpp also targets cellular pathways beyond transcription, which include translation, nucleotide synthesis, and DNA replication (7). Globally, the stringent response reshapes cellular physiology and facilitates stress tolerance by repressing growth-associated processes and upregulating protective responses (16–21). In *P.a*, (p)ppGpp is involved in the regulation of quorum sensing, virulence, motility, and antioxidant defenses (including superoxide dismutases and catalases) (10, 14, 22–25). Finally, the stringent response also confers protection against antibiotic-induced lethality through various molecular mechanisms in several species (9, 22, 26–29).

To date, the system-level characterization of the stringent response in Gram-negative bacteria stems primarily from transcriptomic studies in *E. coli* (6, 17, 30, 31)*,* with only a few done in *P.a* (32, 33). Since mRNA and protein expression often correlate only modestly (34, 35), proteomic approaches can capture the downstream effects of (p)ppGpp at the protein level to better understand (p)ppGpp-dependent phenotypes. Yet, proteomic studies of the stringent response remain scarce (30, 31, 36), with none having been reported in *P.a* to date.

In this study, we performed whole-cell shotgun proteomics using LC-MS/MS to compare a (p)ppGpp-null mutant (9), which carries deletions of *relA* and *spoT* (referred to as ΔSR mutant), to its isogenic wild-type PAO1 parent strain during late exponential and stationary phases in rich medium. Complementary RNA-seq analyses were performed to provide an integrated view of (p)ppGpp-dependent responses. The proteomic data revealed extensive remodeling of cellular processes in the ΔSR mutant that are most pronounced in stationary-phase cells. Moreover, many proteins displayed differential abundance patterns incongruent with their corresponding transcript levels. Transcriptomic analyses also revealed differential expression of several sRNAs that could mediate post-transcriptional regulation of certain stringent response-dependent adaptations. Phenotypic validation of the ΔSR proteome confirmed multiple defects in antioxidant defenses and dysregulated pyoverdine biosynthesis.

## MATERIALS AND METHODS

### Bacterial strains and growth conditions

The *P.a* reference strain PAO1 wild-type (WT) parental strain, its isogenic Δ*relA* Δ*spoT* (ΔSR) mutant with in-frame deletions of *relA* and *spoT*, and the ΔSR mutant complemented with chromosomally integrated *relA* and *spoT* ORFs (ΔSR+SR) were constructed as previously described (9) and confirmed by whole-genome sequencing. Strains were streaked from frozen stock on LB Miller (BD Difco) 1.5% agar. All bacterial cultures were grown as follows unless specified otherwise. Five milliliters of LB Miller medium, inoculated with single colonies, was incubated at 37°C with shaking at 250 RPM for 4–6 h before dilution to an OD_600_ of 0.05 in 25 mL LB Miller medium (250 mL flasks). Cultures were then grown at 37°C with shaking at 200 RPM and sampled at 4 h (late exponential [“Exp”] phase—OD_600_ ~2.0) and at 16–18 h (late stationary [“Stat”] phase—OD_600_ ~4.5–5.0) for proteomic, RNA-seq, and phenotypic analyses. Each sample represents an independent biological replicate, and each experiment was done with at least three biological replicates. For the WT and ΔSR strains, proteomic samples (*n* = 6 biological replicates) were generated from two independent experiments, then processed and analyzed by LC-MS/MS in two separate batches.

### Mass spectrometry sample preparation and analysis

Details of the sample preparation, processing, and analysis by LC-MS/MS are provided in the Supplemental Methods. Briefly, pelleted cells harvested from Exp and Stat cultures (grown as described above) were lysed with lysis buffer, boiled, sonicated, and centrifuged to collect the supernatant containing soluble proteins. Proteins were digested with trypsin, processed, and analyzed by LC-MS/MS.

### Mass spectrometry data processing and analysis

The proteomic data processing workflow is presented in Fig. S1. Peaks from the raw data were aligned to the *Pseudomonas aeruginosa* (strain ATCC 15692/DSM 22644/CIP 104116/JCM 14847/LMG 12228/1C/PRS 101/PAO1) proteome (37) (Proteome ID: UP000002438), and underwent label-free quantification and normalization using MaxQuant (version MQ2.4.4.0) (38, 39). Group-specific parameters included trypsin digestion, standard type (no label), multiplicity of 1 to set label-free quantification (LFQ), and an LFQ minimum ratio count of 1 (40). “Match-between-runs” was selected, assigning peptide identifications from one run to another where a peak is observed based on retention time and *m*/*z* in the MS1 spectrum but not properly identified.

A total of 2,351 proteins were initially identified from ~20,000 peptides. Low-confidence proteins identified by a single unique peptide were filtered out, leaving 2,244 high-confidence proteins identified by at least two unique peptides. Subsequent analysis was performed on log2-transformed data using Perseus (version 2.0.5.0) software platform (41, 42). The R plugin ComBat (R version ≥ 3.5.0) was used to remove batch effects (42, 43). After batch correction, missing values (MV) were imputed using a left-censored imputation method, replacing all MV with a log2 value of 10, which represents a non-zero value lower than any observed value in our data set (44, 45). Replicates were visualized for clustering using a PCA plot. To compare the numbers of identified proteins for each group, we used data prior to MV imputation and generated Venn diagrams using InteractiVenn (46).

To identify differentially abundant proteins (DAP), the data set were split based on growth phase (Exp and Stat). Data from the six biological replicates were pooled for each experimental group, and proteins detected in at least four out of six replicates in at least one group were retained. This threshold was applied to minimize bias due to proteins with low and/or inconsistent detection. A Welch’s *T*-test was performed in Perseus to determine the difference in protein abundance of the ΔSR relative to WT, and those displaying an absolute log2 fold change (|log2 FC|) ≥1 with a Welch’s *T*-test *q*-value (Benjamini-Hochberg FDR) ≤0.05 were classified as significant DAPs.

### Functional analyses of proteomic data

Activation analysis to identify significantly activated or repressed gene ontology (GO) terms was performed with the log2 fold change (FC) value of all identified proteins using the online pathogen-specific Shiny app ESKAPE Act Plus (47). Enriched GO terms were deemed significantly activated or repressed based on an FDR-adjusted *P* value ≤ 0.05 using a binomial test.

Clusters of Orthologous Genes (COGs) annotations and subcellular locations were obtained from the *Pseudomonas* Genome Database (https://pseudomonas.com/) (48). The differentially abundant proteome composition was visualized using the Proteomaps software (https://proteomaps.net/) (49), which relies on KEGG Pathways gene classification. DAPs were also analyzed with TIGRFAM annotations (50), which infer function based on sequence-relatedness (51, 52). DAP and incongruent protein-mRNA pairs were analyzed for GO term pathway enrichment and network clustering using StringDB (https://string-db.org) (53), as detailed in the Supplemental Methods.

### RNA-seq sample preparation and data analysis

At time points matching the proteomic analyses, ~3 × 10^9^ cells were washed in sterile phosphate-buffered saline and resuspended in DNA/RNA Shield (R1100, Zymo Research) for storage until RNA extraction. Details of RNA extraction, library preparation, sequencing, and read mapping are provided in the Supplemental Methods. Following read mapping, gene counts were normalized using DEseq2 (version 1.30.1) (54), log-transformed (log2), and missing values were imputed with 0.5 (low constant). The differential mRNA expression (log2 FC) of the ΔSR relative to WT was calculated for Exp and Stat phases.

### mRNA and protein congruency analysis

All proteins with detectable abundance and corresponding mRNA were included in a correlation analysis between mRNA and protein. The differential abundance of each protein and corresponding mRNA (log2 FC of ΔSR relative to WT) was plotted as a correlation plot and analyzed using linear regression. Protein-mRNA pairs were classified as incongruent when the corresponding protein and mRNA changes occurred in opposite directions (i.e., one with a positive and the other with a negative log2 fold change). Differential gene expression and protein abundance data for each protein-mRNA pair are available in Table S4 (Exp phase) and Table S5 (Stat phase), and the analysis of incongruent protein-mRNA pairs is described in the Supplemental Methods. Reverse-transcriptase quantitative PCR (RT-qPCR) was performed on stationary-phase cells to confirm sRNA transcript levels as described in the Supplemental Methods.

### Computational prediction of the sRNA binding targets of PrrF1 and PrrF2

We used CopraRNA (Freiburg RNA tools) (55, 56) to predict the sRNA binding targets of PrrF1 and PrrF2, as described in the Supplemental Methods.

### Phenotypic assays and PvdA measurements

Stationary-phase cells were analyzed for total intracellular glutathione by high-performance liquid chromatography, for superoxide dismutase activity using the ferricytochrome C assay, for susceptibility to hydrogen peroxide, tert-butyl hydroperoxide, and paraquat oxidants using disc diffusion assays, and for pyoverdine production by fluorescence measurement. PvdA abundance was quantified by western blot. All methods are described in the Supplemental Methods.

### Statistical analysis

Comparisons between ∆SR and WT were done using a Welch’s *T*-test or a multiple *T*-test with Welch’s correction as indicated. Statistical analyses and linear regressions (protein-mRNA correlations) were performed in GraphPad Prism 10 or the indicated tool as specified. Statistical significance was determined based on *P* values noted as *P* ≤ 0.05 (*), *P* ≤ 0.01 (**), *P* ≤ 0.001 (***), *P* ≤ 0.0001 (****).

## RESULTS

### Proteomic profiling of wild-type and ΔSR strains in late exponential and stationary phases

In this study, we characterized the whole-cell proteomes (excluding the secretome) of the PAO1 wild type (WT), the (p)ppGpp-null Δ*relA* Δ*spoT* mutant (ΔSR), and the ΔSR complemented with *relA* and *spoT in trans* (ΔSR+SR) under conditions of planktonic growth in late exponential (Exp) and stationary (Stat) phases in rich LB media (Fig. S2). The sampling time and growth conditions were selected based on previously observed differences in gene expression and phenotypes of the ΔSR mutant (15, 22, 23). With a genome that encodes 5,586 annotated coding sequences (37), our shotgun label-free proteomic analyses detected a total of 2,244 high-confidence proteins, ranging from 1,906 to 2,071 proteins per experimental condition.

**Fig 1:**
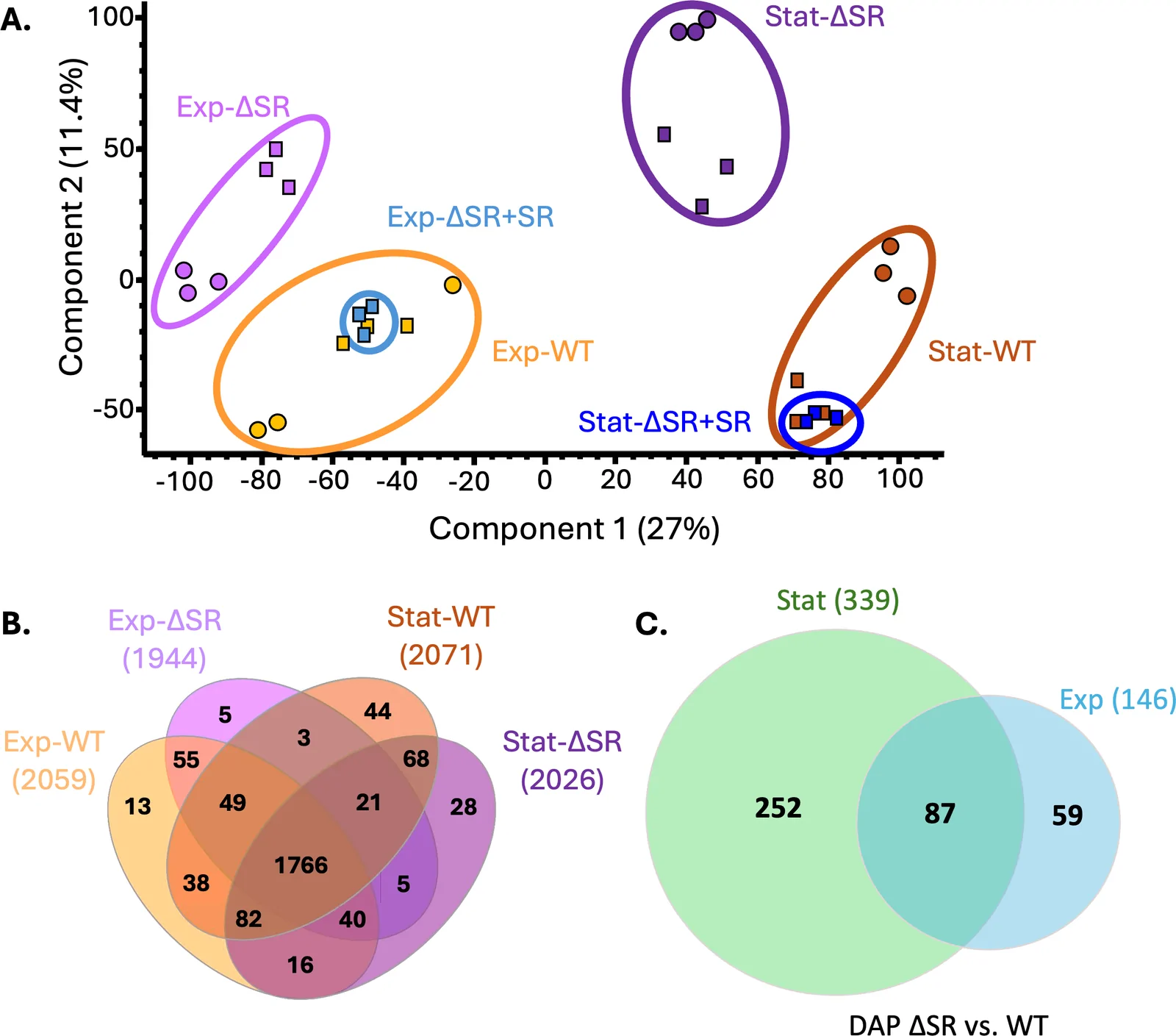
Proteomic profiling of the *relA spoT* mutant (ΔSR) and wild-type (WT) PAO1 in Exp and Stat phases. (**A**) PCA plot of the proteomic profiles for the WT and ΔSR strains in late exponential (Exp) and stationary (Stat) phases. Samples of the WT and ΔSR strains were analyzed as six biological replicates from two independent batches (squares; batch 1, circles; batch 2). The ΔSR+SR strain was included as the complementation control and was only included in three biological replicates in batch 1. (**B**) Venn diagram of all detected proteins in the WT and ΔSR samples for late exponential (Exp) and stationary (Stat) phases. (**C**) Venn diagram of the DAP in Stat or Exp phases. DAPs were defined as an absolute log2 fold change (|log2 FC|) ≥1 in protein abundance in the ΔSR mutant compared to WT and a Benjamini-Hochberg FDR-adjusted *P* value (i.e., Welch’s *T*-test *q*-value) ≤ 0.05.

To visualize the clustering of proteomic profiles across experimental conditions, we performed PCA of all biological replicate samples from the six experimental conditions, namely Exp-WT, Exp-ΔSR, Exp-ΔSR+SR, Stat-WT, Stat-ΔSR, and Stat-ΔSR+SR. As shown in Fig. 1A, PCA revealed the greatest separation between the Exp and Stat conditions. The ΔSR and WT strains were also clearly separated in both growth phases, although batch effects were noted, even after batch correction. Notably, the separation between the ΔSR mutant and WT strains was greater in the Stat than in Exp conditions, as may be predicted since (p)ppGpp accumulates to higher levels during stationary phase (32). As expected, the complemented strain ΔSR+SR clustered with the WT in both growth phases, thus confirming that differences in the proteomic profile of the ΔSR compared to WT were restored upon genetic complementation of *relA* and *spoT*.

Globally, the majority of proteins (1,766 of 2,244 total) were detected in all conditions (Fig. 1B), and >90% of detected proteins were common to Exp and Stat phase cells when comparing each strain to itself (Fig. S3A). During the Exp phase, 1,910 proteins were common to both strains, with 149 (7.2%) and 34 (1.7%) proteins unique to WT or ΔSR, respectively. This was similar during the Stat phase where 1,937 proteins were common, while 134 (6.5%) and 89 (4.4%) proteins were unique to WT or ΔSR, respectively (Fig. S3B). This suggests that widespread physiological changes associated with (p)ppGpp are linked to changes in protein abundance rather than expression of different sets of proteins.

### Functional characterization and network analysis of the (p)ppGpp-dependent proteome

**Fig 2:**
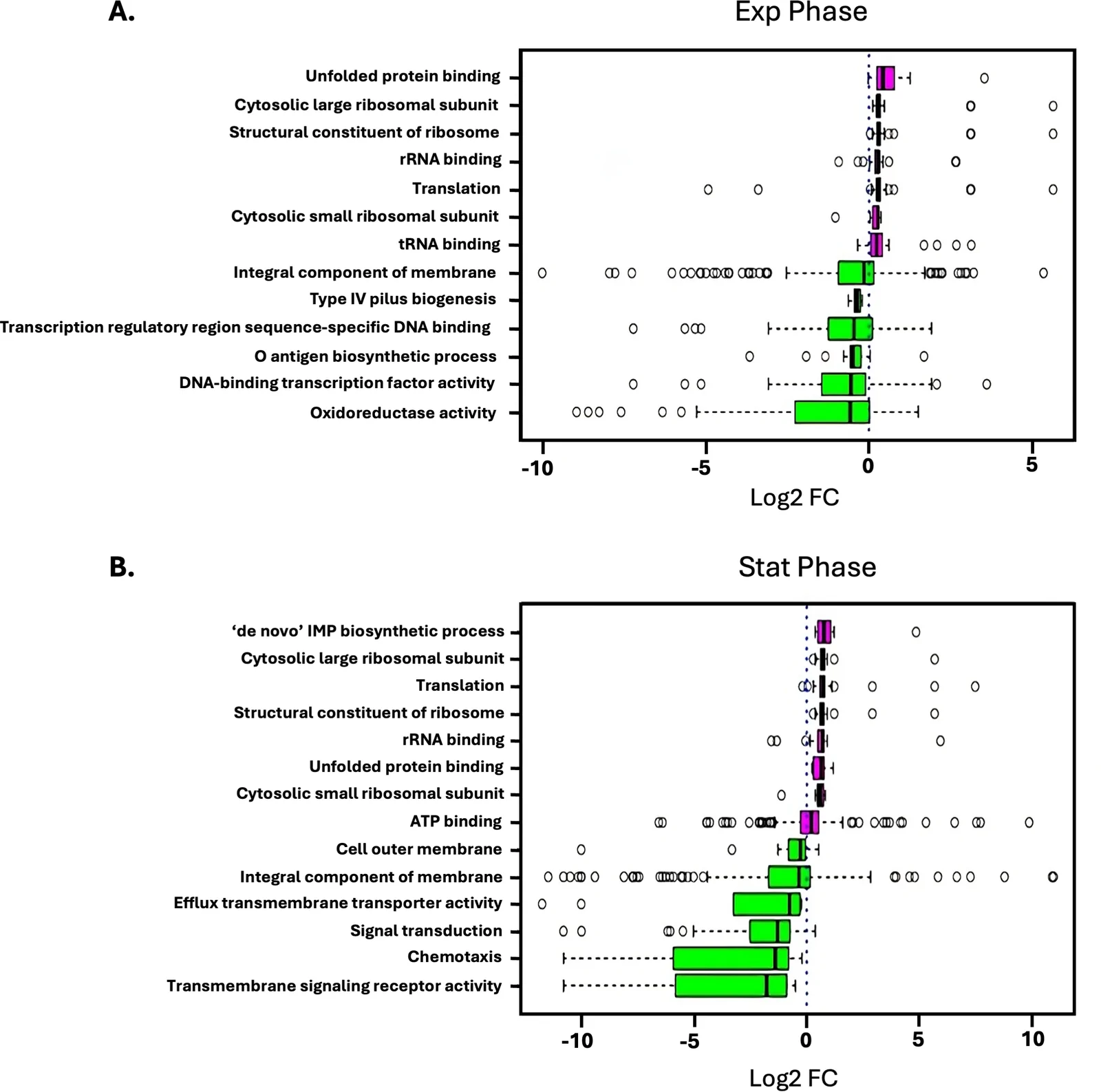
Pathway activation analysis of the ΔSR vs WT proteome. The complete proteomes of the ΔSR mutant are compared to WT for (**A**) Exp or (**B**) Stat phases using ESKAPE Act Plus pathway activation analysis to identify significantly activated or repressed GO terms. Boxplots show the log2 FC of proteins associated with significantly enriched GO terms. Proteins activated in the ΔSR (log2 FC > 0) are shown in magenta, and proteins repressed in the ΔSR (log2 FC < 0) are shown in green. Black bars in boxes represent the median log2 FC.

**Fig 3:**
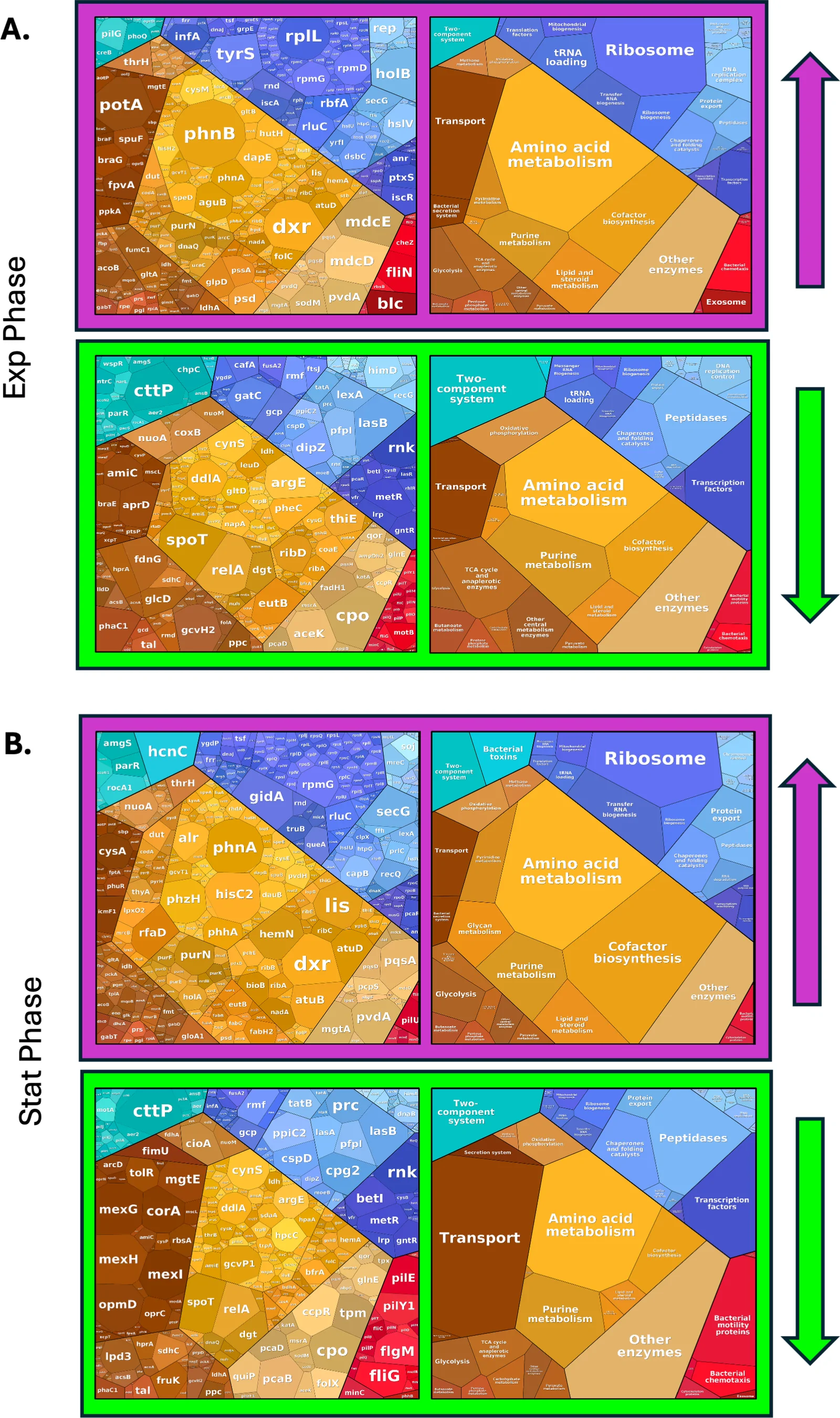
Proteomaps analysis of the ΔSR vs WT proteome. The complete proteome in (**A**) Exp or (**B**) Stat phase data sets is visualized based on KEGG pathway categorization using Proteomap. Proteins are separated into those with increased (magenta) or reduced (green) abundance in the ΔSR compared to WT. Left panels show individual proteins; right panels show functional characterization at the third level of KEGG pathway classifications. Each polygon area reflects protein abundance weighted by protein size.

To gain insight into the cellular processes affected by (p)ppGpp, we first calculated the differential abundance (log2 FC) of all proteins detected in the ΔSR relative to the WT, and performed a systems-level pathway activation analysis for both the Exp and Stat data sets. We used ESKAPE Act Plus (47), a biological pathway analysis tool designed for prokaryotes, to identify significantly activated or repressed GO terms in the ΔSR compared to WT. This revealed that the absence of (p)ppGpp, in both growth phases, led to the activation of processes primarily implicated in protein translation and biosynthesis (Fig. 2A and B). Among the processes repressed in the ΔSR, we observed a greater diversity of functions. In Exp phase cells, cell envelope components (“integral component of membrane” and “O-antigen biosynthetic process”), “type IV pilus biogenesis,” “oxidoreductase activity,” and transcriptional regulatory processes (“transcription regulatory region specific DNA binding” and “DNA binding transcription factor activity”) were repressed (Fig. 2A). In Stat phase, repressed processes largely fell under two major categories: cell envelope components and activity (including “cell outer membrane,” “integral component of membrane,” and “efflux transmembrane transporter activity”) and environmental responses (“signal transduction,” “chemotaxis,” and “transmembrane signaling receptor activity”) (Fig. 2B). Proteomaps analyses (Fig. 3A and B), which allow for visualization of differential abundance of all proteins (ΔSR relative to WT) based on KEGG pathways categorization, were largely consistent with the GO terms ESKAPE Act Plus analyses. They highlighted the large proportion of protein synthesis and ribosomal proteins among those with higher abundance in the ΔSR mutant but also revealed activation of purine biosynthesis, a process known to be regulated by (p)ppGpp in *E. coli* (57).

Next, we compared the ΔSR to the WT proteome in each growth phase for DAP, defined as a difference in protein abundance of the ΔSR relative to WT of at least twofold (|log2 FC| ≥ 1) and with a Welch’s *T*-test *q*-value ≤ 0.05. The batch-corrected, filtered, and imputed LFQ intensities, log2 FC, and statistics for all proteins in Exp and Stat phases are presented in Tables S1 and S2. There were a total of 339 DAP in the Stat phase (Fig. 1C), which represented a 2.3-fold greater number of DAP compared to the Exp phase (which had 146 DAP). Eighty-seven were common to both growth phases.

**Fig 4:**
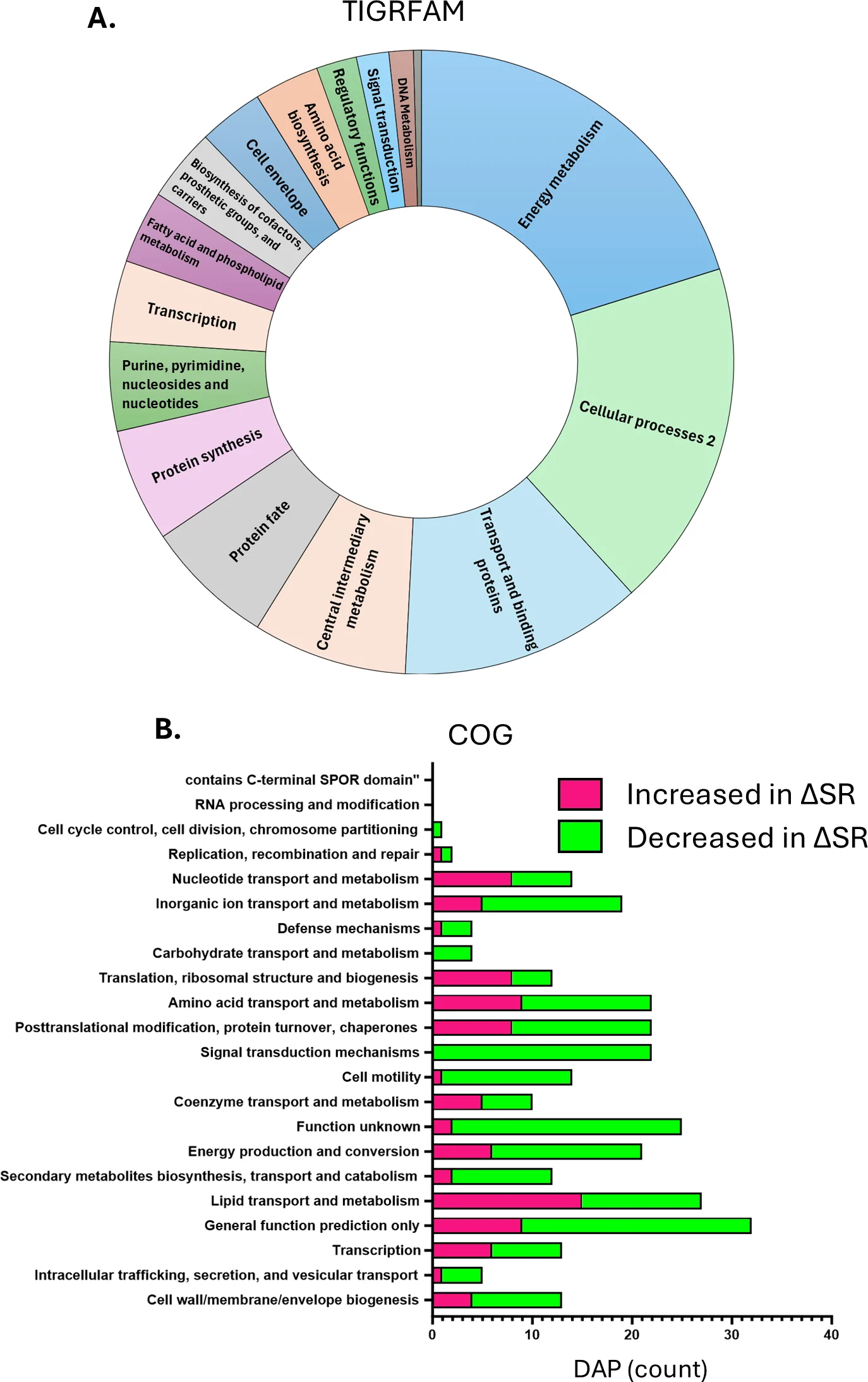
Functional characterization of stationary-phase DAP in the ΔSR relative to WT. (**A**) The “main role” of 238 of 338 (~70%) DAP with known TIGRFAM classification is shown. (**B**) DAP are categorized based on COG classification. Proteins increased (magenta) or reduced (green) in abundance in the ΔSR compared to WT are shown. The complete TIGRFAM classification of DAP from Exp and Stat data sets is available in Table S3.

Given that (p)ppGpp is a major orchestrator of physiological adaptations and stress responses in stationary-phase cells, we focused on the functional characterization of the 339 Stat phase DAP using TIGRFAM annotations (50, 51, 58), which provided functional insights on 70% (238 of 339) of these DAP. As shown in Fig. 4A, 51% of Stat phase DAP fell within three major roles, namely, “Energy Metabolism” (20.2%), consisting mainly of TCA cycle and cellular respiration proteins; “Cellular Processes 2” (18.1%), consisting of chemotaxis transducers, quorum sensing, and stress response proteins (e.g., OsmC, SodM, AhpC); and “Transport and binding proteins” (12.6%), consisting of ion and transmembrane transporters. Other roles encompassed biosynthesis and metabolism of various macromolecules, cell envelope, and regulatory functions (complete data available in Table S3).

Among the Stat phase DAPs, 219 were decreased in abundance in the ΔSR relative to WT, while 120 had increased abundance, indicating that (p)ppGpp more broadly maintains or induces cellular functions than it represses them during stationary phase. To understand the physiological remodeling resulting from (p)ppGpp, DAP were grouped by COG annotations (Fig. 4B). Core metabolic and transport categories (including nucleotide, inorganic ion, amino acid, and lipid) displayed bidirectional changes, consistent with extensive metabolic reprogramming in the absence of (p)ppGpp. In contrast, proteins associated with “cell motility,” “signal transduction,” and “secondary metabolites biosynthesis, transport*,* and catabolism” categories were predominantly reduced in the ΔSR mutant, suggesting a role for the stringent response in inducing or maintaining these adaptive functions. Only three categories—“nucleotide transport and metabolism,” “lipid transport and metabolism,” and “translation ribosomal structure and biogenesis”—showed a predominance of increased abundance in the ΔSR mutant, indicating that (p)ppGpp acts to repress these processes.

**Fig 5:**
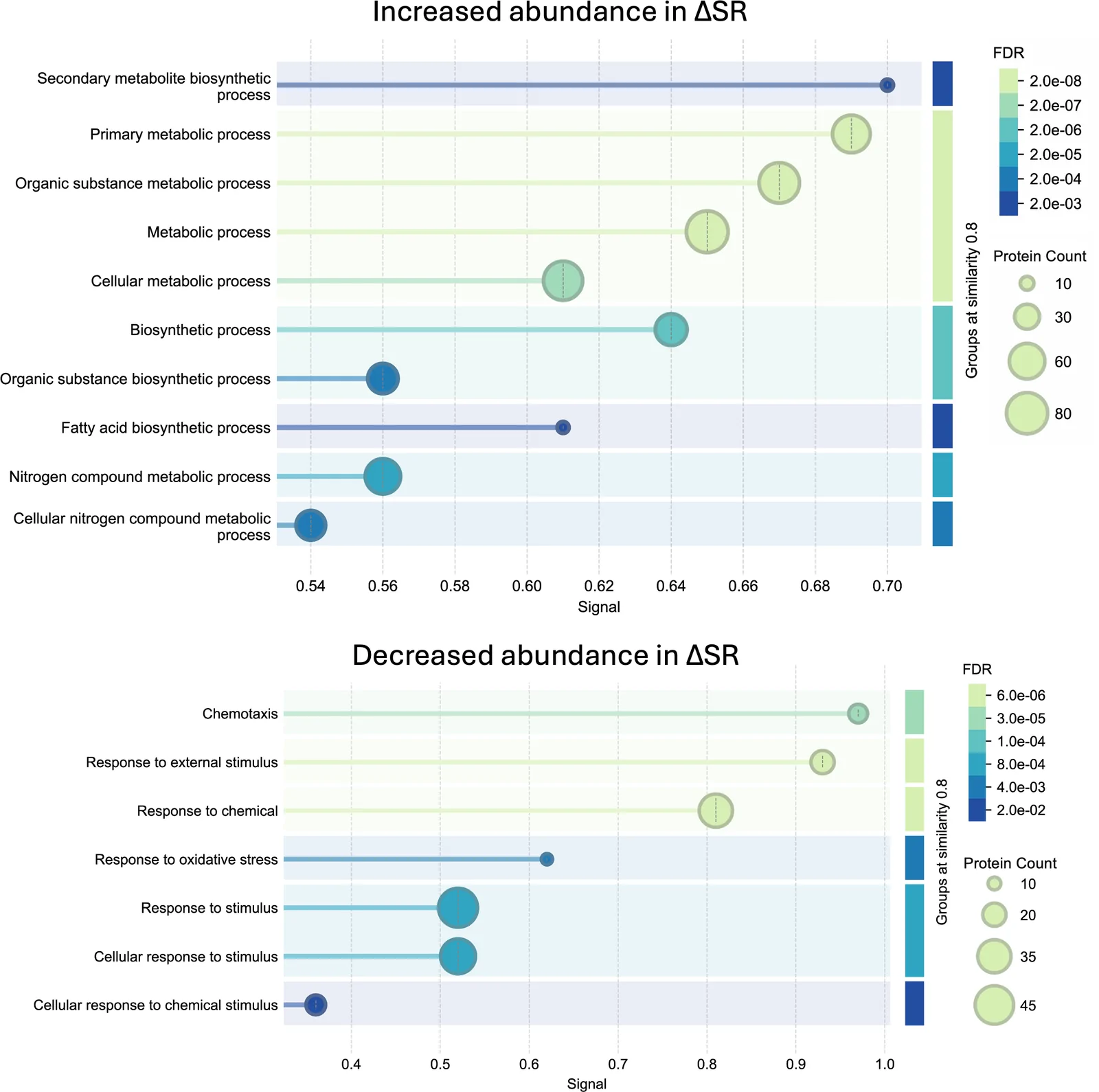
GO term enrichment analysis of biological processes for stationary-phase DAP in the ΔSR relative to WT. Enrichment analysis of the 120 higher-abundance DAP (top) and the 219 lower-abundance DAP (bottom) in Stat phase using STRING DB. The *x*-axis displays the signal value, which considers the number of observed vs expected proteins for the enriched term and the −log(FDR). The enriched terms (*y*-axis) are grouped based on term similarity >0.8. The size of the circles represents the total number of proteins in each enriched term. The color of each circle is dictated by the FDR value. Data were selected based on minimal thresholds: FDR ≤ 0.05, signal ≥ 0.25, and strength ≥ 0.25.

To define the biological processes most affected by (p)ppGpp during stationary phase, we performed GO term enrichment analysis of Stat-phase DAP (Fig. 5). DAP with higher abundance in the ΔSR mutant were significantly enriched for a broad range of biosynthetic and metabolic processes, including tRNA, fatty acid, and lipids. In contrast, proteins with reduced abundance in ΔSR were enriched for chemotaxis and stimulus response pathways, notably those related to oxidative stress.

**Fig 6:**
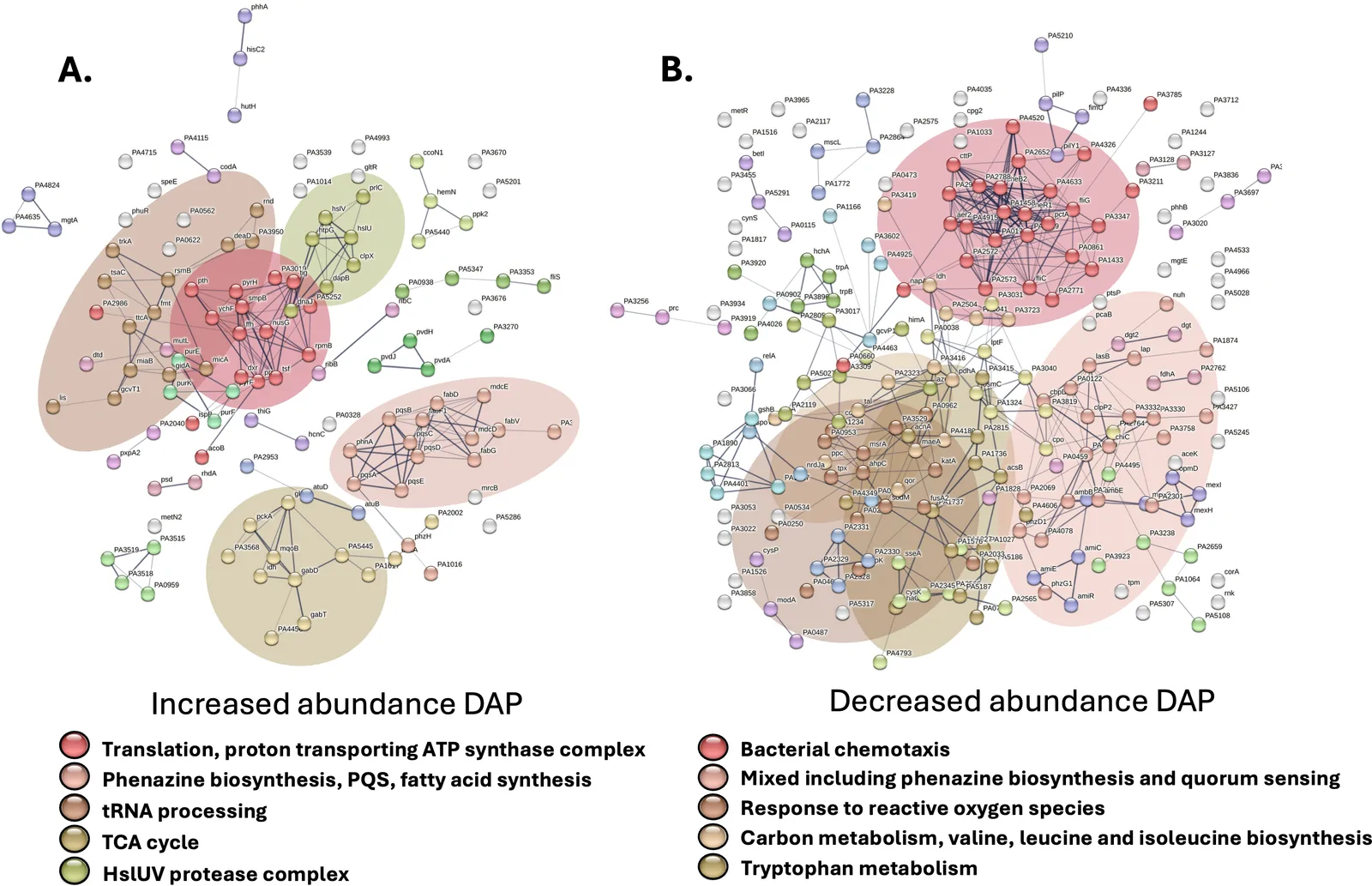
Network analysis of stationary-phase DAP in the ΔSR relative to WT. Proteins with (**A**) higher abundance and (**B**) lower abundance in ∆SR relative to WT were analyzed for clustering using the MCL algorithm in StringDB (inflation parameter 1.8). The top five largest clusters are highlighted on the network maps. Nodes’ colors are based on assignment to each cluster. The width and opaqueness of the edges between nodes represent the confidence of the interaction between proteins (String score). Confidence represents the strength of available data supporting the interaction between proteins. The complete data for all MCL clusters is available in Table S10.

Next, we performed STRING (53) network analysis, followed by Markov clustering (MCL), to resolve the system-level organization of these changes. This approach identified several clusters of highly interconnected proteins among Stat phase DAP (complete data available in Table S10). Those with higher abundance in the ΔSR mutant formed 19 clusters (Fig. 6A), with the largest consisting of proteins involved in translation and ATP synthase complex (*n* = 16 DAP), phenazine biosynthesis (*n* = 15 DAP), tRNA processing (*n* = 13 DAP), and TCA cycle (*n* = 12 DAP). DAP with lower abundance in the ΔSR mutant formed 26 clusters (Fig. 6B), with the largest clusters consisting of proteins involved in chemotaxis (*n* = 27 DAP), phenazine biosynthesis, and quorum sensing (*n* = 20 DAP), responses to reactive oxygen species (*n* = 15 DAP), carbon and branched amino acid metabolism (two clusters of *n* = 14 DAP each).

Together, these analyses indicate that (p)ppGpp drives a coordinated remodeling of the proteome during stationary phase, repressing core metabolic and biosynthetic functions—including translation, nucleotide, and lipid metabolism—while inducing or maintaining pathways involved in chemotaxis, environmental sensing, adaptation, and detoxification, particularly oxidative stress responses.

### Incongruences between the proteomes and transcriptomes suggest possible (p)ppGpp-dependent post-transcriptional regulation of adaptive responses in stationary phase

**Fig 7:**
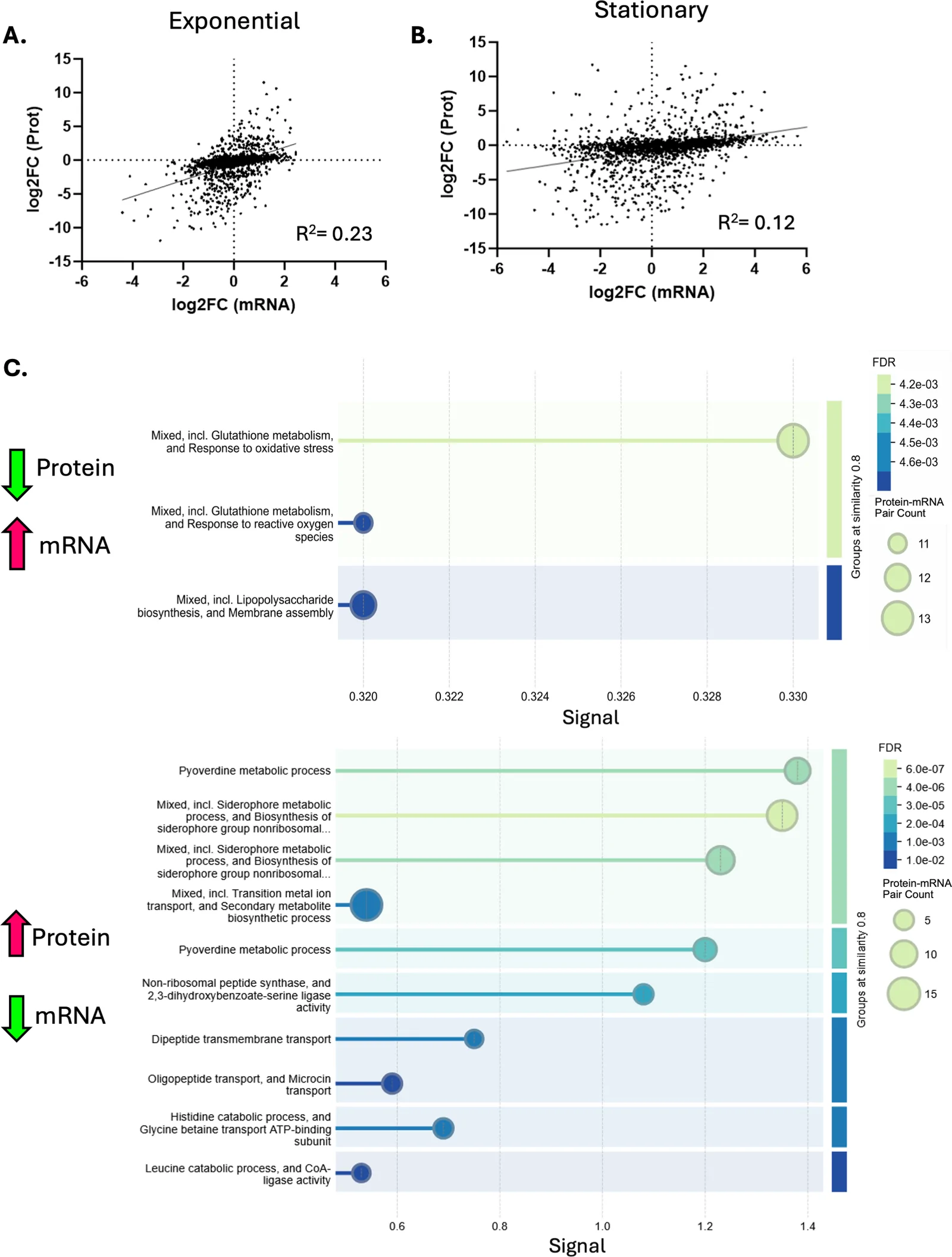
Correlation and incongruency analysis between protein and mRNA responses in the ΔSR relative to WT. The correlation between the log2 FC (ΔSR vs WT) of protein abundance and the corresponding log2 FC of mRNA expression in the (**A**) Exp phase or (**B**) Stat phase is shown in scatter plots. *R*^2^ = coefficient of determination from the linear regression. (**C**) STRING local network cluster enrichment analysis of the incongruent protein-mRNA pairs in Stat phase. The analysis was performed on protein-mRNA pairs where incongruence was deemed significant based on a differential protein or mRNA change of at least twofold (|log2 FC| > 1) in the ΔSR relative to WT. This represents 283 proteins with reduced DAP but up-expressed mRNA response (top panel) and 112 proteins with higher abundance DAP but down-expressed mRNA (bottom panel). The *x*-axis displays the signal value for the enriched network clusters, which considers the number of observed vs expected proteins per enriched STRING cluster, as well as the −log(FDR). Enriched clusters (*y*-axis) are grouped based on term similarity >0.8. The size of the circles represents the total number of protein-mRNA pairs in each enriched term.

Transcriptomic profiling imparts invaluable insights into cellular states and responses, and many studies have been performed under various experimental conditions to understand the role of (p)ppGpp signaling on microbial physiology (59–63). However, it may overlook post-transcriptional and translational regulatory mechanisms that cells utilize to modulate protein expression. Since (p)ppGpp exerts a multilayered direct and indirect control of transcription and translation (18), we sought to investigate the relationship between (p)ppGpp-dependent protein abundance and mRNA expression. We performed transcriptome sequencing using the same culture conditions as the proteomic analyses and compared the differential abundance (ΔSR relative to WT) of each protein to the differential expression of their respective mRNA (the complete data sets are available in Tables S4 and S5). Consistent with other reports (64), the correlation between the protein and mRNA responses was weak in both Exp and Stat phases (*R*^2^ = 0.23 and 0.12, respectively, Fig. 7A and B), albeit worse in the Stat phase. If the comparisons were limited to the significant DAP (defined as |log2 FC| ≥ 1 and adjusted *P* value ≤ 0.05), the correlation between DAP and differential gene expression improved moderately (*R*^2^ = 0.38 for Exp and 0.21 for Stat phase) but remained lower in Stat phase cells (Fig. S4).

Next, we assessed the correspondence between mRNA expression and protein abundance by classifying protein-mRNA pairs as congruent when their ΔSR vs WT log2 fold changes were directionally aligned (both positive or both negative), regardless of the fold change magnitude. Conversely, protein-mRNA pairs were considered incongruent when their responses directionally diverged, defined as opposite-sign log2 fold changes (one positive and one negative). Recognizing that protein abundance lags behind transcriptional changes, we reasoned that such directionally discordant responses could represent post-transcriptional or translational regulation. We thus compared the differential response for each protein-mRNA pair and found that 30.8% (536/1,742) and 33.7% (601/1,780) showed incongruent expression in Exp and Stat phases, respectively (Tables S4 and S5). By limiting to those with at least twofold change (|log2 FC| ≥ 1) in protein abundance or mRNA expression, 149 exponential phase and 395 stationary phase protein-mRNA pairs showed incongruent expression. To explore whether the genes with incongruent protein-mRNA expression in stationary phase are enriched in specific pathways or processes, we performed a STRING pathway enrichment analysis (Fig. 7C). This revealed that genes with higher abundant proteins but down-expressed mRNA in the ΔSR mutant were enriched for pyoverdine and siderophore synthesis (e.g., *pvdA/N*), secondary metabolite and non-ribosomal peptide biosynthetic processes, as well as several amino acid and peptide catabolic and transport processes. Genes with reduced protein abundance but up-expressed mRNA were enriched for glutathione metabolism and responses to oxidative stress (e.g., *gshA*, *ahpB/C*, *sodB*, *oxyR*), lipopolysaccharide biosynthesis, and membrane assembly (e.g., *lptD*/*E*).

Interestingly, several genes with incongruent protein-mRNA expression were involved in oxidative stress and envelope stress responses. This raised the possibility of post-transcriptional regulation through non-coding sRNAs, which are known mechanisms to modulate stress responses in Enterobacteriaceae and *P.a* (65, 66). Our RNA-seq analyses revealed that 20+ predicted or known sRNA were differentially expressed in the ∆SR mutant during stationary phase (Table S6). Notably, the expression of PrrF1 and PrrF2, two highly similar sRNA involved in iron homeostasis and virulence (67, 68), was significantly down-expressed in the ∆SR mutant, and the high pyoverdine production in the ΔSR mutant phenocopies that of the PrrF mutant (69). This led us to confirm the transcript levels of PrrF1/2 by RT-qPCR, alongside RsmY and RsmZ, the top differentially expressed sRNA between ∆SR and WT strains (Fig. S5). We then examined which genes with incongruent mRNA-protein differential expression may be direct or indirect PrrF targets. In addition to known PrrF targets (69, 70), computational prediction of sRNA binding targets with CopraRNA (55, 56, 71) suggested that 311 genes are potentially regulated by PrrF1/2 (Table S8). Among these, 112 were identified in our proteomic data sets and 40 putative PrrF1/2 targets showed incongruent protein-mRNA differential expression in the ∆SR mutant, including *sodB*, *cioA*, *leuA*, *dnpA*, and *dnaA*. Other PrrF regulated genes, such as *katA*, were significantly down-regulated but with congruent differential protein and mRNA expression.

### Functional validation of the ΔSR proteome

**Fig 8:**
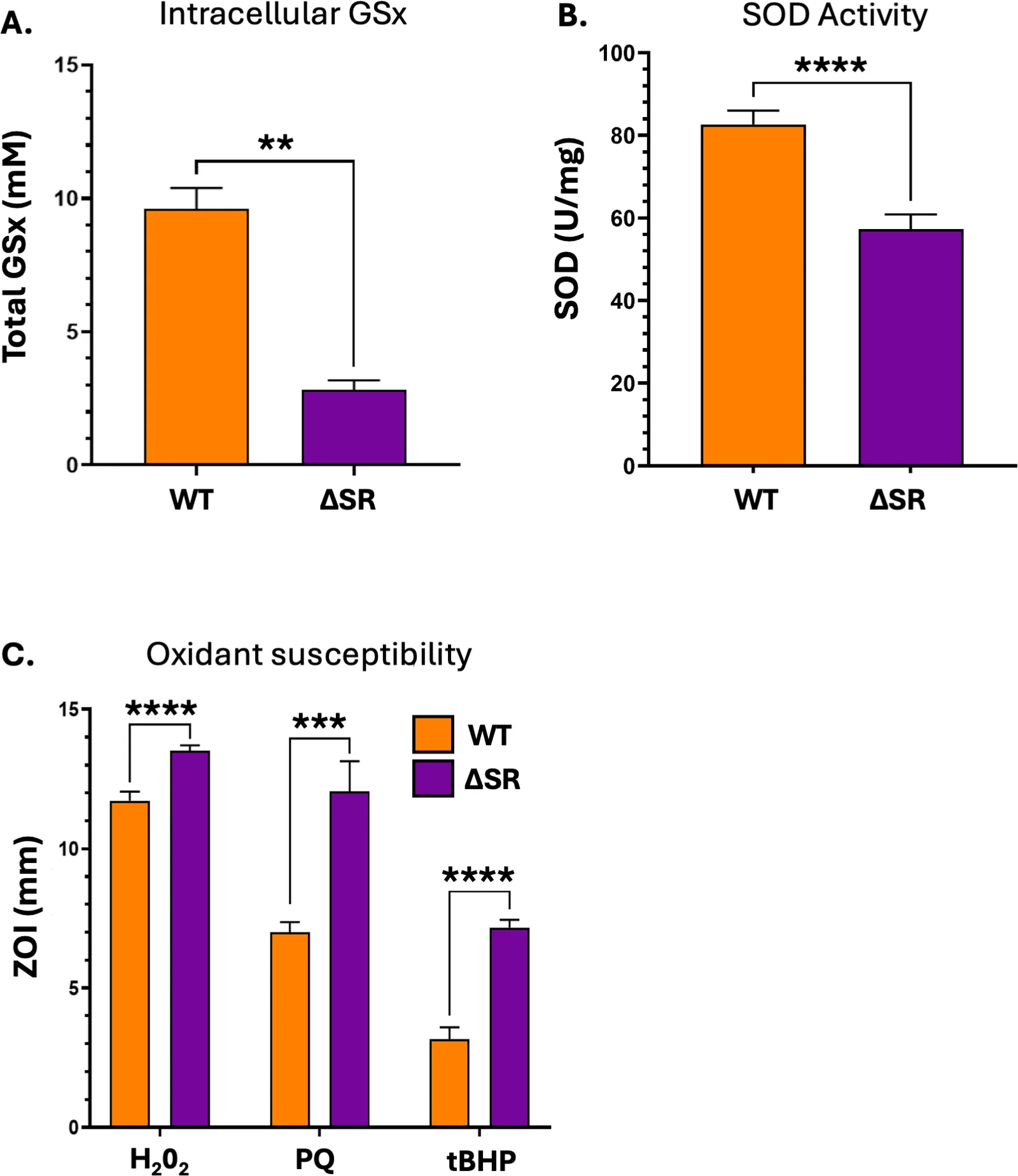
Oxidative stress susceptibility in stationary phase ∆SR mutant and WT. (**A**) Total intracellular glutathione measured by HPLC. (**B**) Superoxide dismutase (SOD) activity measured via inhibition of cytochrome C oxidation assay. (**C**) Susceptibility to 30% hydrogen peroxide (H_2_O_2_), 100 mM paraquat (PQ), or 1.5 µM tert-butyl hydroperoxide (tBHP). ZOI, zone of inhibition. Statistical analysis was done using a Welch’s *T*-test (**A**, **B**) or a multiple comparison *T*-test with Welch’s correction (**C**). *P* > 0.05 (NS), *P* ≤ 0.05 (*), *P* ≤ 0.01 (**), *P* ≤ 0.001 (***), *P* ≤ 0.0001 (****).

**Fig 9:**
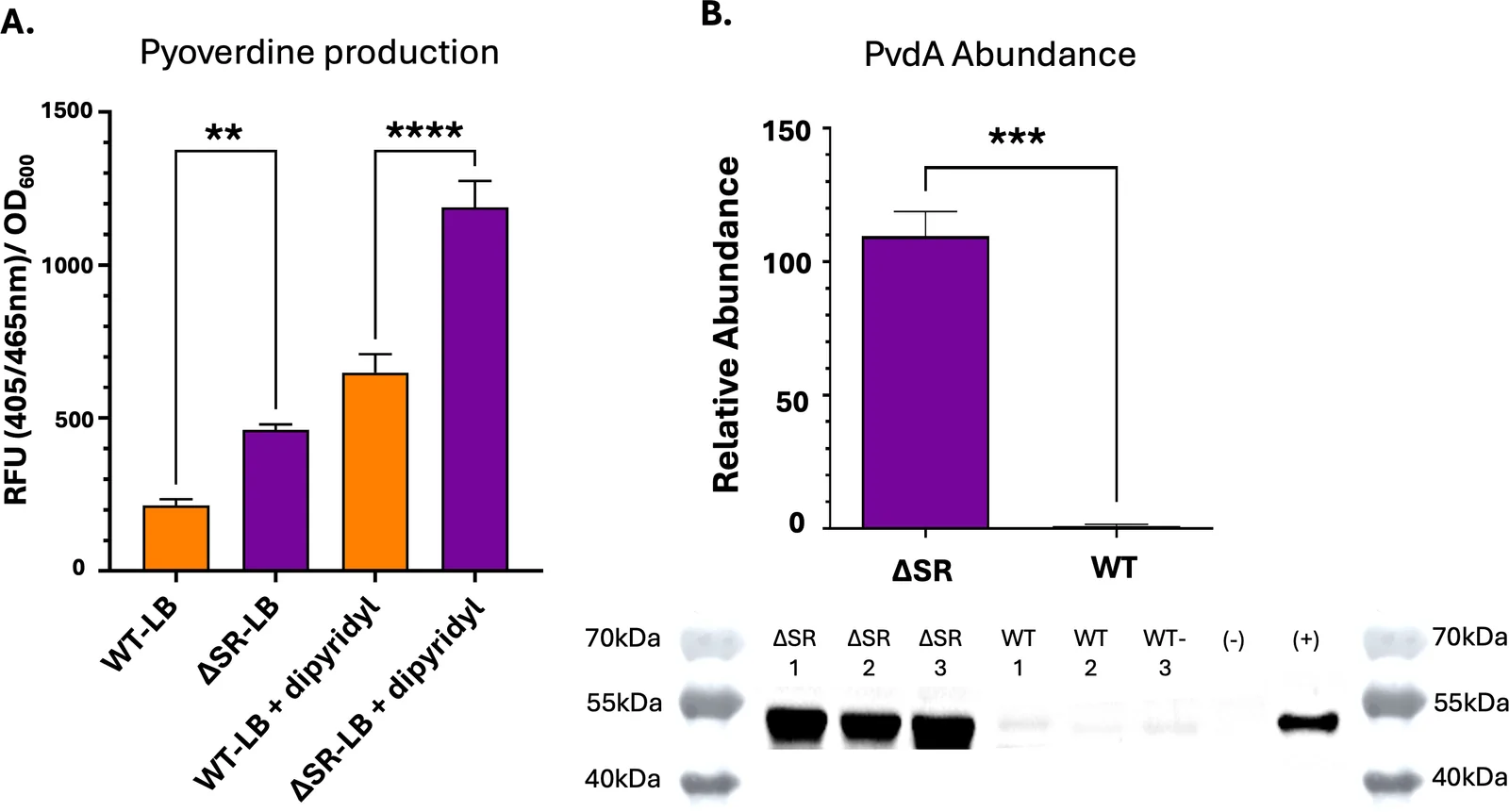
PvdA abundance and pyoverdine production in stationary-phase ∆SR mutant and WT. (**A**) Pyoverdine levels in stationary-phase WT and ∆SR cultures in iron-replete (LB) and iron-limited (LB + 0.25 mM 2,2′-dipyridyl) conditions, measured as relative fluorescence at Ex 405 nm/Em 465 nm. Results are shown as mean (± SD) from *n* = 3 biological replicates. (**B**) PvdA protein abundance measured by western blot from stationary-phase WT and ∆SR mutant cultures. PvdA abundance was quantified by band densitometry and is shown as mean (± SD) from three independent biological replicates, each quantified by three independent blots. The image shows the PvdA bands (50 kD) for each sample (30 µg protein from cell lysates per lane) alongside the *pvdA* mutant sample (negative control) and 200 ng purified recombinant PvdA (positive control). One of three representative blots is shown, and the full blot image is available in Fig. S8. Statistical comparison was done using one-way ANOVA and Holm-Sidak multiple comparisons test for panel **A** and unpaired *T*-test for panel **B**. *P* value ≤ 0.01 (**), 0.001 (***), and 0.0001 (****).

We sought to validate several key differences of the stationary phase ΔSR proteome compared to WT using functional phenotypic assays and western blot. First, we confirmed that ΔSR cells have reduced glutathione levels (Fig. 8A), a finding likely attributable to reduced GshA and GashB, the γ-glutamylcysteine synthetase and glutathione synthetase required for glutathione biosynthesis. The ΔSR mutant also harbors reduced superoxide dismutase activity (Fig. 8B), increased susceptibility to paraquat, hydrogen peroxide, and tert-alkyl hydroperoxide (Fig. 8C), findings that support reduced abundance of alkyl-hydroperoxide reductases AhpB/C, superoxide dismutases SodB/M, and catalase KatA. Second, the abundance of PvdA/N, the pyoverdine biosynthesis proteins, was validated by measurement of pyoverdine production and PvdA by western blot. Consistent with enrichment of pyoverdine biosynthesis proteins among the overabundant stationary-phase DAP, pyoverdine production is 1.8–2.2-fold higher in the ΔSR mutant in both iron-replete and iron-limited conditions in stationary phase (Fig. 9A; Fig. S7). PvdA abundance in stationary-phase cells, measured by western blot, was also over 100-fold higher in the ΔSR compared to WT (Fig. 9B; Fig. S8). Finally, we note that our group and others have also previously shown that the ΔSR mutant produced higher PQS (15) and lower LasR-regulated LasB (14), consistent with the DAP identified in our STRING network analyses.

## DISCUSSION

Our study represents the first proteomic data set to explore the effect of the stringent response in *P.a* and provides a comprehensive view of its effect in reshaping bacterial physiology and inducing stress responses during the stationary phase. Our results also highlight the differences between the proteome and transcriptome, including the potential indirect role of post-transcriptional regulation via sRNA, such as PrrF1/2, in (p)ppGpp-dependent metabolic and stress adaptations.

While shotgun proteomics provides unbiased broad coverage of the proteome, we recognize that our study presents inherent limitations. First, protein detection may be incomplete due to inefficient cell lysis, peptide digestion, or poor ionization. Nonetheless, we identified a total of ~1,700–2,300 high-quality proteins, a coverage comparable to other studies in *P.a* (72–76). Additionally, membrane proteins are difficult to extract due to relatively low abundance and insolubility (77, 78). Although our data raised the possibility that cytoplasmic membrane proteins may be underrepresented relative to the complete reference proteome (Fig. S6), their proportions in our study (~13%–15% of all proteins) were comparable to others in *P.a* (76, 79). In fact, the coverage achieved is challenging to assess since the true number of membrane proteins expressed under specific conditions is unknown. Finally, we chose to compare cultures in late exponential phase to those in late stationary phase to capture previously observed functional and expression differences between the WT and ΔSR mutant strains (15, 22, 23). However, we recognize that the selected time points do not fully capture the dynamic changes across growth phases. Consequently, the DAP identified here likely underestimates the full scope of (p)ppGpp-dependent regulation. Expanding the analysis to additional time points or stress conditions would undoubtedly uncover additional proteins and functions regulated by (p)ppGpp.

Functional characterizations and network analyses of the proteomic data sets confirmed that (p)ppGpp regulation has pleiotropic effects on many biosynthetic and metabolic pathways in both exponential and stationary phase *P.a*. In the absence of (p)ppGpp, stationary phase *P.a* cells fail to repress biosynthetic processes, including translation, nucleotide, and lipid metabolism, and do not respond appropriately to stress and stimuli, via pathways such as chemotaxis, iron homeostasis, and oxidative stress responses. This leaves cells vulnerable to external stressors, such as oxidative or antibiotic damage (22, 23). Key aspects of the ΔSR mutant proteome were validated in our study through phenotypic assays and western blots that corroborated the defects in antioxidant defenses (glutathione biosynthesis, superoxide dismutase [SOD], and alkyl-hydroperoxide reductases) and dysregulated pyoverdine biosynthesis. Our proteomic findings are also consistent with the functional defects previously observed by our group (9) and others (10, 24) in the *relA spoT Pa* mutant, including reduced pyocyanin and protease production, impaired biofilm production, and motility.

Our global proteome analysis suggests several global similarities between the stringent response in *E. coli* and *P.a*. Consistent with our findings, failure to induce the stringent response leads to dysregulation of translation, purine biosynthesis, response to stress, and chemotaxis proteins in *E. coli* (30, 31, 57). Additionally, transcriptomic studies of the *E. coli* stringent response mutants also show dysregulation of several transcription factors and signal transduction pathways (80). However, there are several important differences in the regulatory networks of *P.a* and *E. coli* that likely impact the indirect or downstream effects of (p)ppGpp. For example, the control of RpoS expression, one of the major mediators of the stringent response, differs in *E. coli* and *P.a*, including (p)ppGpp-dependent mechanisms (81). Furthermore, RpoS is required for the general stress response in *E. coli*, particularly survival to nutrient starvation, oxidative stress, and extremes of pH and temperature (82). In *P.a*, although RpoS affects the expression of over 700 genes, its protective role for stress survival is less significant than in *E. coli*, and its relationship with Vfr and quorum sensing likely accounts for its role in modulating virulence and pathogenesis (83, 84).

The link between (p)ppGpp and oxidative stress sensitivity has been shown in several bacterial species (85–88), including *P.a* in previous work by our group (9, 22, 23). This study highlights the extensive effect of (p)ppGpp on oxidative stress responses at the proteomic level, with altered abundance of superoxide dismutases, catalases, alkyl hydroperoxidases, and glutathione metabolism among others. These oxidative stress defects likely result from complex interactions between (p)ppGpp and global regulators (e.g., RpoS [89], quorum sensing [14, 32], or transcriptional regulators, such as OxyR [11] or MexT [24]). Recognizing that our study focused on the reference strain PAO1, the effect of the stringent response on stress responses may differ in other *Pa* strains where the regulatory network hierarchy differs.

While numerous transcriptomic studies have previously investigated the global contribution of (p)ppGpp to the physiological state and adaptation of bacteria (11, 90–92), very few proteomic studies have investigated this (30, 31, 93). This led us to examine the relationship between matched mRNA expression and protein abundance in the WT and ΔSR mutant strains in both exponential and stationary phases. The low correlation between the mRNA and protein responses is consistent with other studies in *P.a*, including studies that examined discrepancies in different growth phases (94, 95). We note that comparing mRNA and protein abundance measured at the same time points does not account for temporal delays between transcription and translation; nevertheless, such comparisons can be informative, as they highlight genes under post-transcriptional or post-translational control. In our study, we defined incongruent expression as cases where the direction of change in protein abundance was opposite to that of the corresponding mRNA transcript. We therefore reasoned that protein-mRNA pairs exhibiting incongruent responses may represent (p)ppGpp-dependent regulatory mechanisms acting post-transcriptionally—such as mRNA stability (96), modulation of translation efficiency (97, 98), ribosome activity (99), protein degradation (100), or regulation by non-coding sRNA.

Bacteria frequently use non-coding sRNA for rapid responses to stress. However, very few studies to date have reported on stringent response-regulated sRNA (101, 102). Our transcriptomic analyses showed differential expression of several regulatory non-coding sRNAs in the ΔSR mutant relative to WT, including PrrF1/2 and RsmY/Z. PrrF1 and PrrF2, two highly similar sRNAs, are functional homologs of RyhB that play critical roles in regulating iron homeostasis in *P.a*, particularly the biosynthesis of pyoverdine, a key siderophore involved in iron acquisition and virulence (67, 68). The reduced PrrF1/2 expression in the ΔSR mutant suggests that dysregulation of these sRNAs may underlie the aberrant iron homeostatic responses, including the increased expression of the pyoverdine biosynthetic cluster, a phenotype also reported in the PrrF mutant (69). PrrF1 and PrrF2 have complex direct and indirect mechanisms of post-transcriptional control, which include not only base-pairing and destabilization of mRNA transcripts (67) but also interactions with Hfq (68), regulation of a third sRNA PrrH, and modulation of PQS (69). Whether the observed changes in the ΔSR mutant are due to direct or indirect effects thus remains to be determined. Interestingly, Argaman et al*.* also showed that the (p)ppGpp synthase RelA indirectly stimulates the activity of RyhB (102), an sRNA that regulates iron homeostasis in *E. coli* in response to iron limitation (103, 104). Such results parallel our findings, which highlight the role of the stringent response in modulating sRNAs responsive to iron limitation.

In addition, the ΔSR mutant had reduced expression of RsmY and RsmZ, two sRNAs that antagonize the translational repressor RsmA/N and modulate quorum sensing as part of the Gac/Rsm pathway that regulates motility, biofilm, and virulence expression (105). Reduced expression of RsmY/Z in the ΔSR mutant may contribute to its dysregulated quorum sensing and motility, but their exact contribution remains to be validated given the complex regulatory cross-talk and network-level control underlying these processes (106).

This study defines the broad impact of (p)ppGpp on the *P.a* PAO1 proteome, revealing extensive remodeling that is most pronounced during stationary phase. The stringent response primarily acts to repress broad metabolic and biosynthetic processes while sustaining pathways linked to chemotaxis, quorum sensing, and stress adaptation. This study also revealed (p)ppGpp’s effects on sRNA expression and instances of protein-mRNA incongruence, suggesting post-transcriptional control. These findings highlight the complementary value of proteomic profiling to capture the full scope of bacterial adaptive responses and underscore the multilayered role of (p)ppGpp in coordinating the transition to stationary phase.

## Data Availability

The mass spectrometry raw data and output tables are available online on the ProteomeXchange Consortium via the PRIDE (107) partner repository (data set identifier PXD067764). RNA-seq reads are available in the NCBI Sequence Read Archive (accession number PRJNA1308619). Whole-genome sequences for the WT and ΔSR strains are available at the NCBI BioProject (ID: PRJNA1489530).

## SUPPLEMENTAL MATERIAL

Supplemental figures msystems.01742-25-s0001.pdf Figures S1 to S6.

Supplemental methods msystems.01742-25-s0002.pdf Additional methods.

Supplemental Tables msystems.01742-25-s0003.xlsx Tables S1 to S10.

ASM does not own the copyrights to Supplemental Material that may be linked to, or accessed through, an article. The authors have granted ASM a non-exclusive, world-wide license to publish the Supplemental Material files. Please contact the corresponding author directly for reuse.
