# Supplementary material for: Proteomic profiling of the Pseudomonas aeruginosa stringent response highlights global adaptive responses during stationary phase
Source: mSystems. 2026 Sep 17;11(10):e01742-25. doi: 10.1128/msystems.01742-25 (PMC13712967; doi:10.1128/msystems.01742-25)
Supplement: Supplementary file 1 [file msystems.01742-25-s0001.pdf]

Proteomic profiling of the *Pseudomonas aeruginosa* stringent response highlights global adaptive responses during stationary phase

Jacquelyn Rich[1], Sam Taylor- Wardell[2], Dorival Martins[1], Malika Khakhimova[1], Gowthami Sampathkumar[1], Tevy Chan[1,4,5], Thi Tuyet Mai Nguyen[6], Geoffrey McKay[6], Lorne Taylor[3], Daniel Pletzer[2], Dao Nguyen[1,5,#]

Supplementary Figures

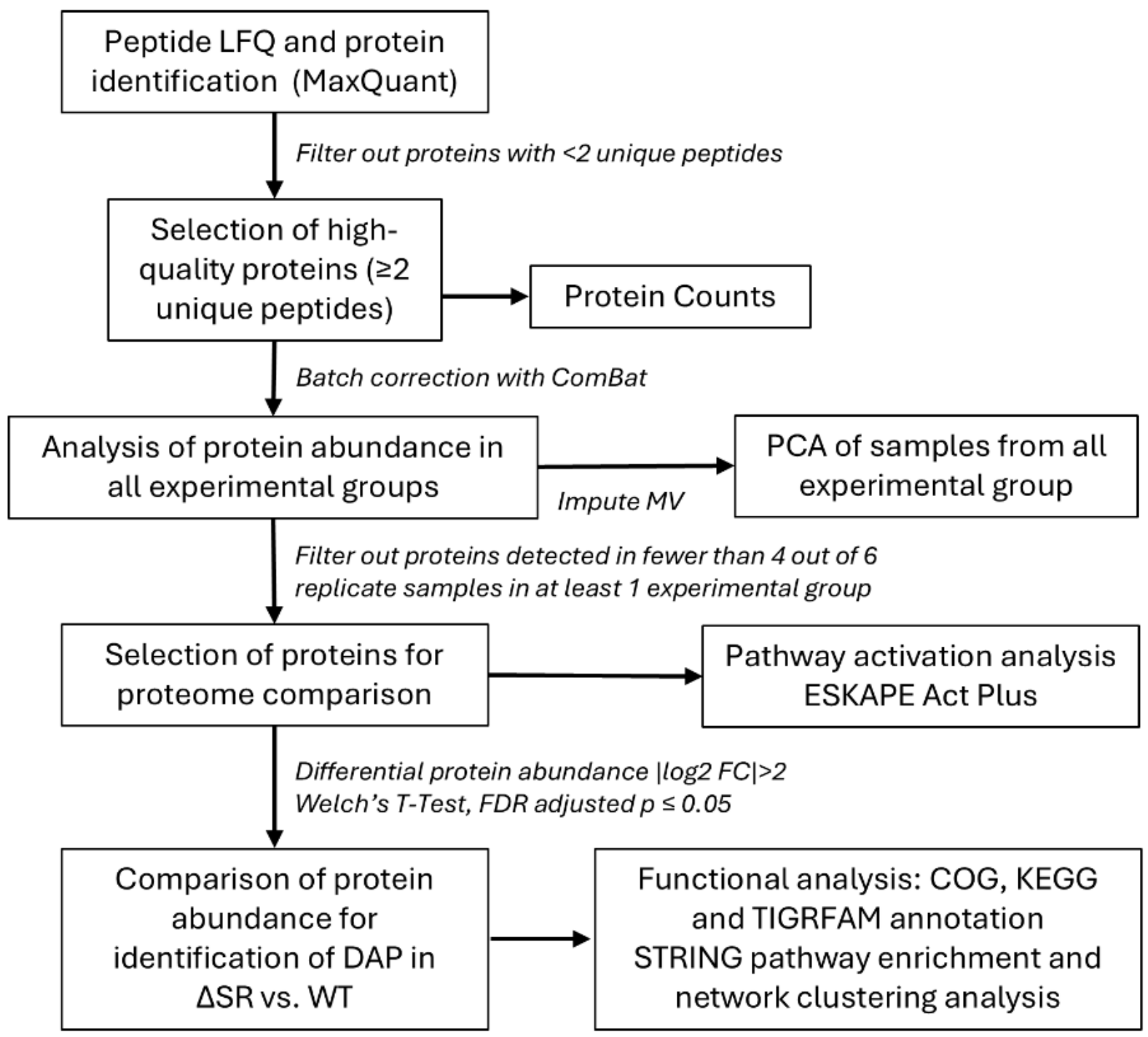

Supplemental Figure S1: Flowchart of proteomic data analysis. MS peak identifications was performed in Maxquant[20,21] and proteins identified by less than 2 unique peptides were filtered out. Batch correction was performed in Perseus[22] using the ComBat R-plugin[23]. Batch corrected and imputed data were used to perform PCA clustering. WT and ΔSR datasets were then split by growth phase, and proteins were filtered out if they were not detected in at least 4 of 6 biological replicates for at least one group. Statistical analysis using a Welch's T-Test with a Benjamini-Hochberg FDR = 0.05 was performed in Perseus to identify differentially abundant proteins (DAP).

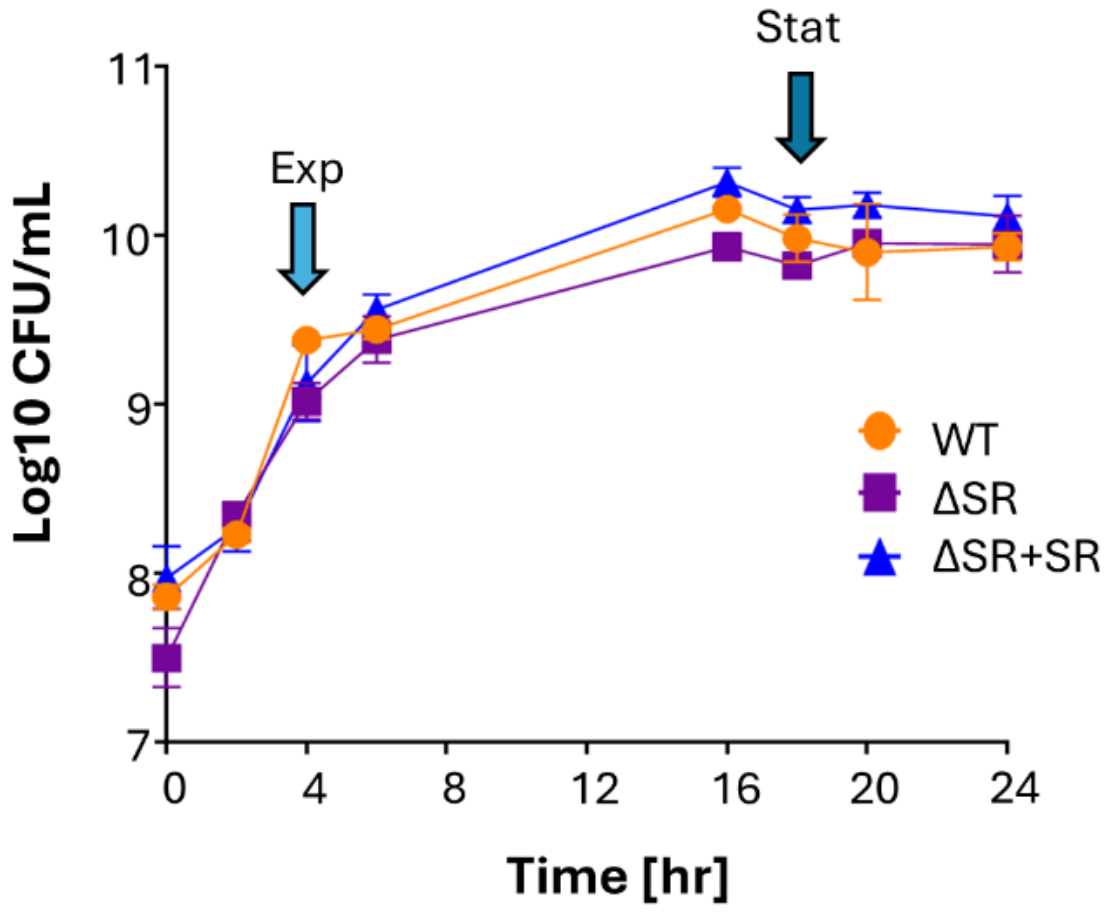

Supplemental Figure S2: Growth curve for each strain used in this study.

Bacteria were grown in 25 mL of LB Miller at 37 °C with shaking at 200 RPM as done for the proteomic and transcriptomic analyses and described in the methods. Arrows indicate the timepoints chosen for Exp and Stat phase samples.

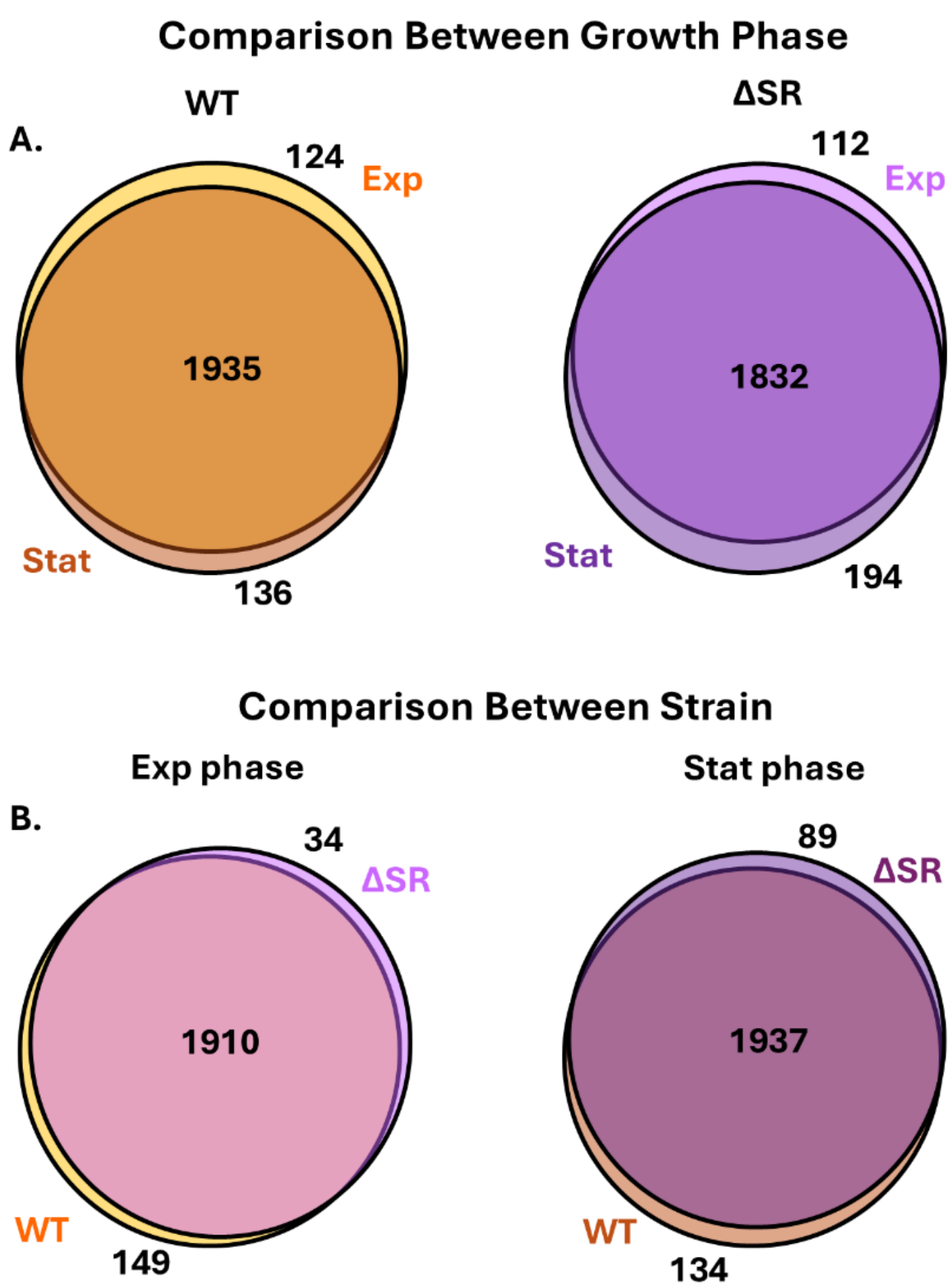

Supplementary Figure S3: Overlap of proteins identified in different experimental groups.

A) Venn diagram of the number of all proteins detected in WT or ΔSR. B) Venn diagram of the number of all proteins detected in exponential (Exp) and stationary (Stat) phase. High quality proteins (with at least 2 unique peptides) are included in this analysis.

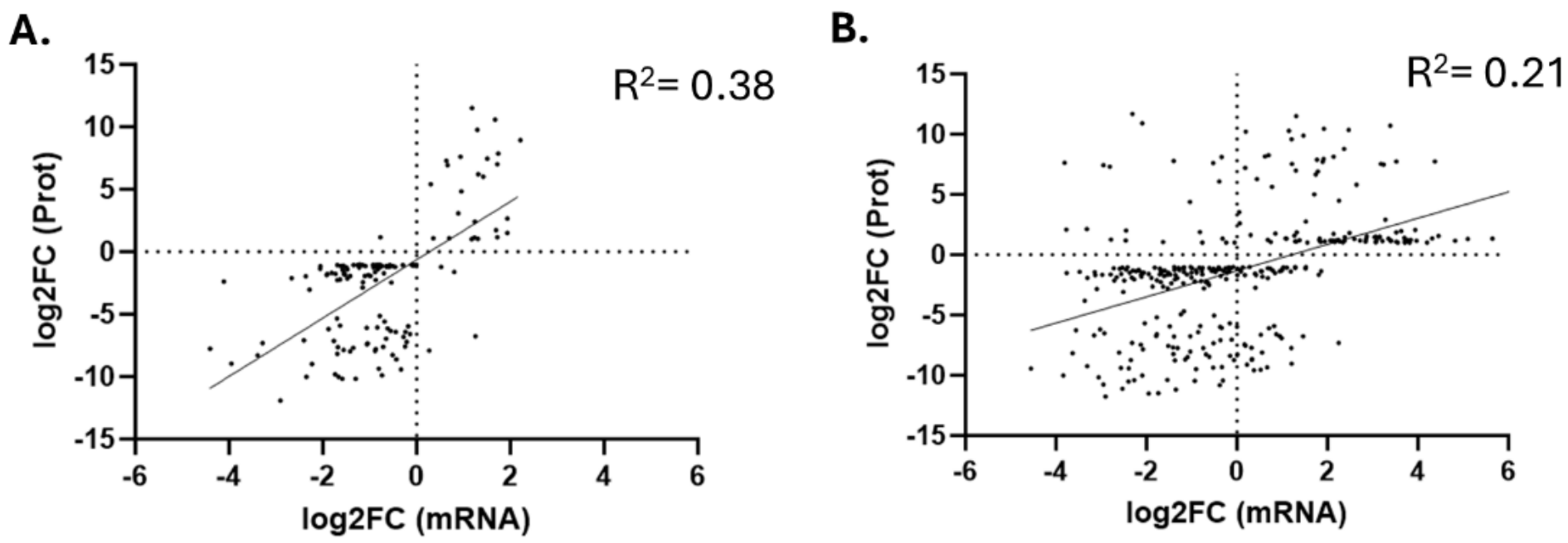

Supplemental Figure S4: Correlation analysis between protein and mRNA responses for proteins with significant differential abundance in the ΔSR relative to WT. The analysis was performed on significant DAP, defined as |log2FC| >1 and FDR adjusted p-value ≤0.05. The correlation between protein log2 FC (ΔSR relative to WT) compared to the corresponding log2 FC mRNA expression is shown in a scatter plots for Exp phase (A) or Stat phase (B). The R^2 coefficient of determination was calculated based on linear regression.

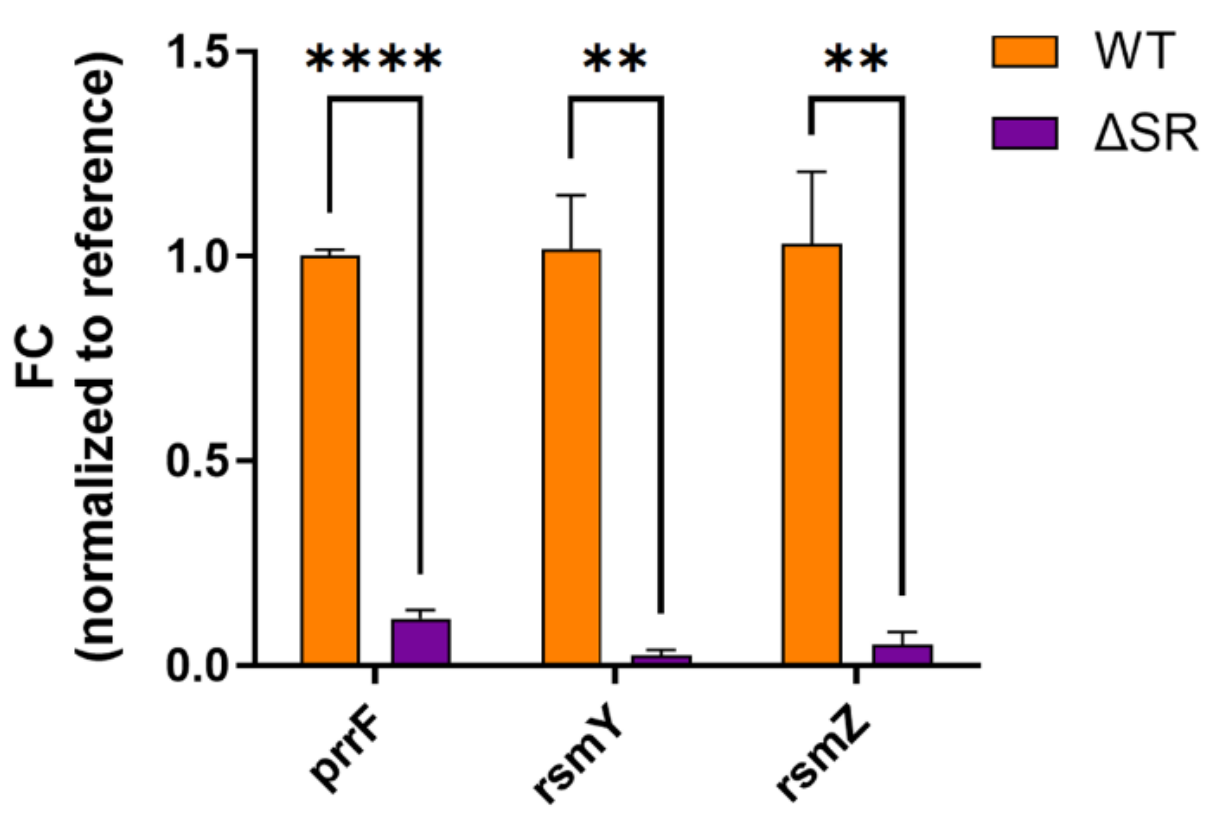

Supplemental Figure S5: sRNA expression in stationary phase WT and ΔSR cells.

Relative transcript levels of non-coding sRNA PrrF1/2, RsmY and RsmZ were measured by RT-qPCR and calculated as fold change (FC) normalized to the geometric mean of two stable reference genes (*proC* and *gyrB*). Results are shown as mean (+/-SD) of n=3 biological replicates, each measured with n=3 technical replicates. Statistical comparison was done with the Holm-Sidak multiple t tests comparing ΔSR to WT. P-value **<0.01, ****<0.0001.

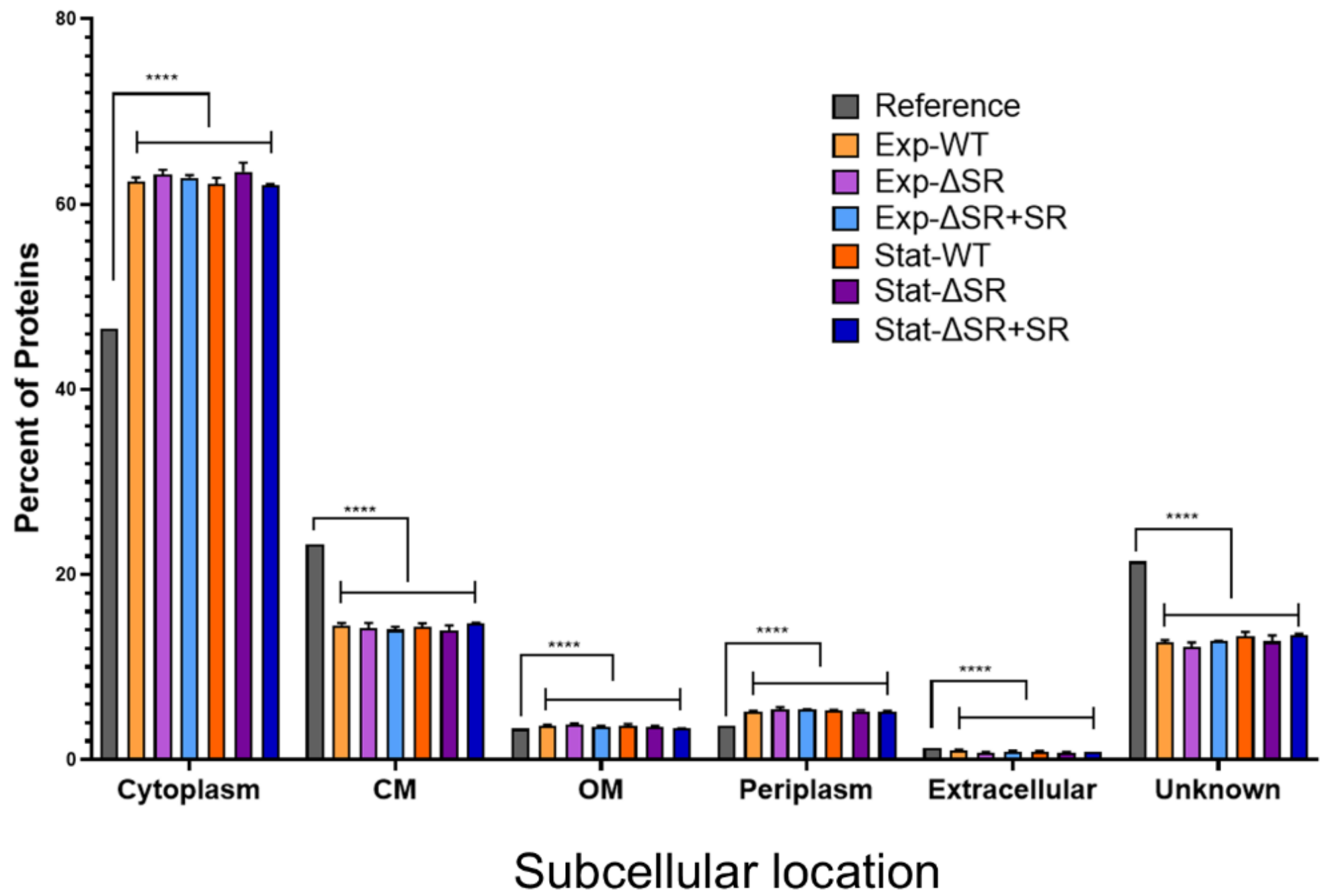

Supplemental Figure S6: Subcellular locations of all detected proteins. All proteins detected in the WT, ΔSR and ΔSR+ SR strains were compared to the PAO1 reference proteome and were assigned a subcellular location based on data available at https://pseudomonas.com/[26]. The categories of "*fimbrial*" and '*flagellar*" were excluded since they represented less than 1% of proteins. CM: cytoplasmic membrane, OM: outer membrane. Statistical comparisons were done using a one Sample T-test comparing the mean of the 6 experimental groups to the reference value. **** P-value <0.0001.

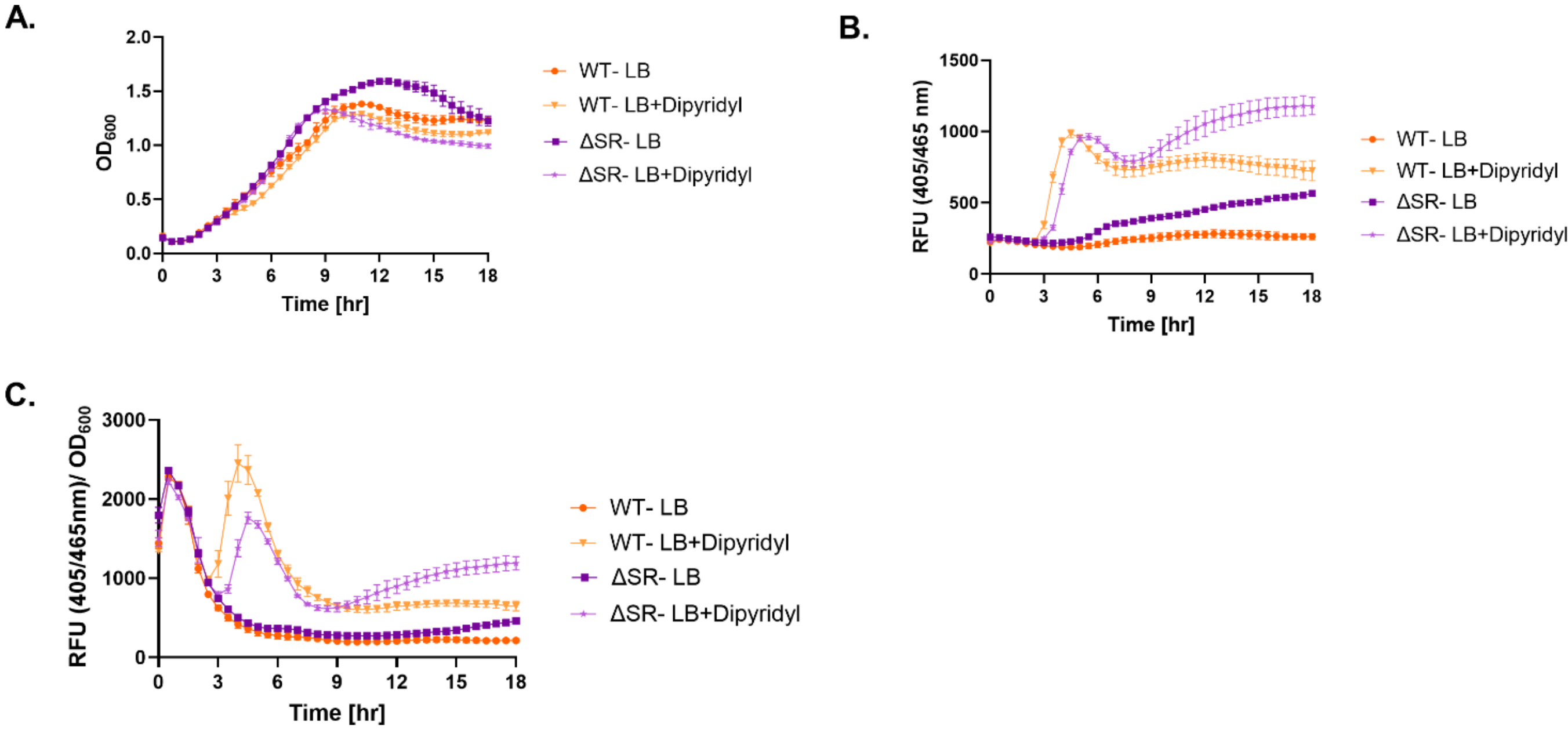

Supplemental Figure S7: Growth curve and pyoverdine abundance of WT and ΔSR in high and low iron conditions. WT and ΔSR grown for 18 hrs in LB Miller (iron replete) or LB Miller supplemented with 0.25 mM 2,2'-dipyridyl (iron deplete). A) OD_{600} for growth; B) fluorescence (RFU) at Ex 405/ Em 465 nm for pyoverdine levels ; C) RFU normalized by OD_{600} for pyoverdine levels normalized to cell density. Results are shown as mean +/-SD from n=3 biological replicates from one representative experiment.

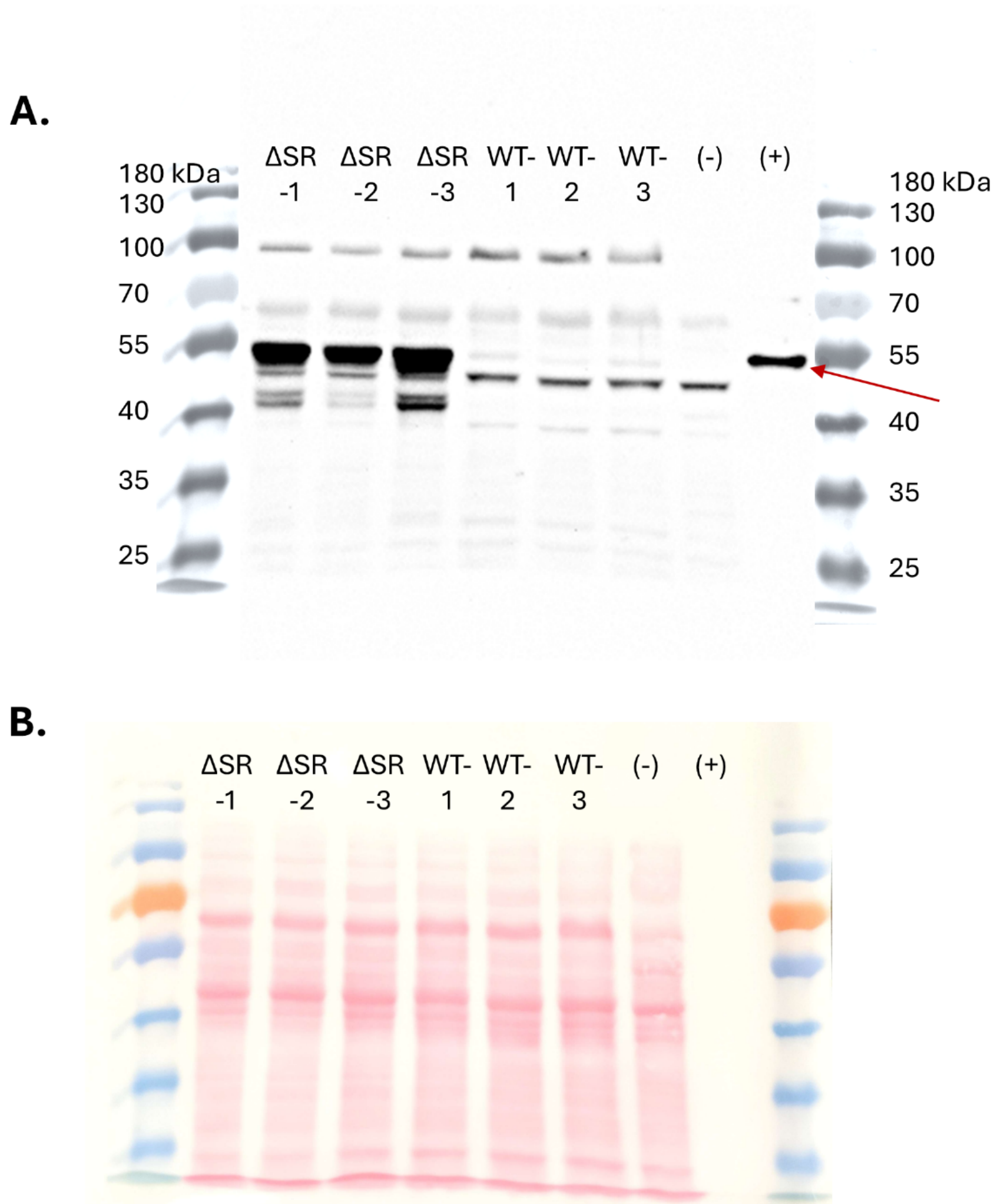

Supplemental Figure S8: Western blot and Ponceau to measure PvdA abundance in the ΔSR and WT strains. Proteins were transferred onto a nitrocellulose membrane. (A) The arrow indicates the ~50-kDa band detected with purified recombinant PvdA (positive + control) and in all WT and ΔSR samples. The *pvdA* knockout mutant strain (negative - control) did not show any detectable 50-kDa band. (B) The same membrane as in A stained with 0.1% Ponceau S to assess transfer efficiency and enable total protein normalization. Densitometric analysis of total protein and PvdA was performed using ImageJ. To calculate fold changes, the PvdA protein signal was normalized to the total protein signal (Ponceau S) for each individual lane, and the resulting values were normalized to the WT sample.

References

1. Koeppen, K., Hampton, T. H., Neff, S. L. & Stanton, B. A. ESKAPE Act Plus: Pathway Activation Analysis for Bacterial Pathogens. *mSystems* **7**, e00468-22 (2022).

2. Szklarczyk, D. *et al.* The STRING database in 2025: protein networks with directionality of regulation. *Nucleic Acids Res* **53**, D730–D737 (2025).

3. Szklarczyk, D. *et al.* STRING v11: protein–protein association networks with increased coverage, supporting functional discovery in genome-wide experimental datasets. *Nucleic Acids Research* **47**, D607–D613 (2019).

4. Stover, C. K. *et al.* Complete genome sequence of Pseudomonas aeruginosa PAO1, an opportunistic pathogen. *Nature* **406**, 959–964 (2000).

5. Dobin, A. *et al.* STAR: ultrafast universal RNA-seq aligner. *Bioinformatics* **29**, 15–21 (2013).

6. Ewels, P., Magnusson, M., Lundin, S. & Käller, M. MultiQC: summarize analysis results for multiple tools and samples in a single report. *Bioinformatics* **32**, 3047–3048 (2016).

7. Wood, D. E., Lu, J. & Langmead, B. Improved metagenomic analysis with Kraken 2. *Genome Biol* **20**, 257 (2019).

8. Kim, C., Jothi, R., Oh, K.-K. & Park, D. S. Novel Species-Specific Primers Enable Accurate Detection and Quantification of *Pseudomonas aeruginosa* via qPCR. *Journal of Food Protection* **88**, 100467 (2025).

9. Janssen, K. H. *et al.* Functional Analyses of the RsmY and RsmZ Small Noncoding Regulatory RNAs in Pseudomonas aeruginosa. *J Bacteriol* **200**, e00736-17 (2018).

10. Chourashi, R., Weiner, J. M., Hoang, T.-M., Ouattara, K. & Oglesby, A. G. The Pseudomonas aeruginosa PrrF sRNAs and PqsA promote biofilm formation at body temperature. *J Bacteriol* **208**, e0050725 (2026).

11. Chervoneva, I. *et al.* Selection of optimal reference genes for normalization in quantitative RT-PCR. *BMC Bioinformatics* **11**, 253 (2010).

12. Vandesompele, J. *et al.* Accurate normalization of real-time quantitative RT-PCR data by geometric averaging of multiple internal control genes. *Genome Biol* **3**, RESEARCH0034 (2002).

13. Bustin, S. A. *et al.* The MIQE guidelines: minimum information for publication of quantitative real-time PCR experiments. *Clin Chem* **55**, 611–622 (2009).

14. Wright, P. R. *et al.* CopraRNA and IntaRNA: predicting small RNA targets, networks and interaction domains. *Nucleic Acids Res* **42**, W119-123 (2014).

15. Wright, P. R. *et al.* Comparative genomics boosts target prediction for bacterial small RNAs. *Proc Natl Acad Sci U S A* **110**, E3487-3496 (2013).

16. Raden, M. *et al.* Freiburg RNA tools: a central online resource for RNA-focused research and teaching. *Nucleic Acids Res* **46**, W25–W29 (2018).

17. Ano, S. *et al.* Inflammation and airway hyperresponsiveness after chlorine exposure are prolonged by Nrf2 deficiency in mice. *Free Radic Biol Med* **102**, 1–15 (2017).

18. Martins, D. *et al.* Superoxide dismutase activity confers (p)ppGpp-mediated antibiotic tolerance to stationary-phase *Pseudomonas aeruginosa*. *Proc Natl Acad Sci USA* **115**, 9797–9802 (2018).

19. Forman, H. J. & Fridovich, I. Superoxide dismutase: a comparison of rate constants. *Arch Biochem Biophys* **158**, 396–400 (1973).

20. Cox, J. *et al.* Accurate proteome-wide label-free quantification by delayed normalization and maximal peptide ratio extraction, termed MaxLFQ. *Mol Cell Proteomics* **13**, 2513–2526 (2014).

21. Tyanova, S., Temu, T. & Cox, J. The MaxQuant computational platform for mass spectrometry-based shotgun proteomics. *Nat Protoc* **11**, 2301–2319 (2016).

22. Tyanova, S. *et al.* The Perseus computational platform for comprehensive analysis of (prote)omics data. *Nat Methods* **13**, 731–740 (2016).

23. Yu, S.-H. *et al.* Expanding the Perseus Software for Omics Data Analysis With Custom Plugins. *Current Protocols in Bioinformatics* **71**, e105 (2020).

24. Haft, D. H. *et al.* TIGRFAMs and Genome Properties in 2013. *Nucleic Acids Research* **41**, D387–D395 (2013).

25. Lewin, G. R. *et al.* Application of a quantitative framework to improve the accuracy of a bacterial infection model. *Proceedings of the National Academy of Sciences* **120**, e2221542120 (2023).

26. Winsor, G. L. *et al.* Enhanced annotations and features for comparing thousands of Pseudomonas genomes in the Pseudomonas genome database. *Nucleic Acids Res* **44**, D646-653 (2016).
