# Supplementary material for: Proteomic profiling of the Pseudomonas aeruginosa stringent response highlights global adaptive responses during stationary phase
Source: mSystems. 2026 Sep 17;11(10):e01742-25. doi: 10.1128/msystems.01742-25 (PMC13712967; doi:10.1128/msystems.01742-25)
Supplement: Supplementary file 2 [file msystems.01742-25-s0002.pdf]

Proteomic profiling of the *Pseudomonas aeruginosa* stringent response highlights global adaptive responses during stationary phase

Jacquelyn Rich[1], Sam Taylor- Wardell[2], Dorival Martins[1], Malika Khakhimova[1], Gowthami Sampathkumar[1], Tevy Chan[1,4,5], Thi Tuyet Mai Nguyen[6], Geoffrey McKay[6], Lorne Taylor[3], Daniel Pletzer[2], Dao Nguyen[1,5,#]

Supplementary Methods

Growth curve

Cultures were reconstituted from glycerol stock on LB agar before inoculating 5 mL of LB Miller media (BD Difco) with single colonies. Subculture was incubated at 37 °C with shaking at 250 RPM for 4-6 hrs before being diluted to an OD_{600} of 0.05 in 25 mL of LB Miller media (250 mL flasks). Aliquots were taken at 0, 2, 4, 6, 8, 16, 18, 20, and 24 hrs, serially diluted in sterile PBS and plated for viable colony forming unit (CFU) counts on LB agar. For growth curves performed to compare pyoverdine production under iron replete or limited conditions, cultures were grown in LB Miller (200 µL in 96 well plates) supplemented with 0.25 mM 2,2'-dipyridyl or DMSO carrier control. Absorbance at OD_{600} was measured every 30 min with continuous shaking at 37 °C using multimode plate reader (BioTek Cytation 5, Agilent).

Mass spectrometry sample preparation and analysis

Cell pellets were collected from 5 mL of exponential and stationary phase cultures following centrifugation (7,000 g x 5 min) and frozen at -20 °C until lysis. Thawed pellets were resuspended in 250 µL of lysis buffer (4% SDS, 0.2% (w/v) deoxycholic acid, 50 mM Tris(2-carboxyethyl) phosphine hydrochloride (TCEP-HCl), 100 mM ammonium bicarbonate, pH 8), then boiled at 90-95 °C for 10 min, vortexing every 2 min. Samples were sonicated on ice (3x 10 sec on/ 10 sec off, 20% amplitude ~100W) and centrifuged for 10 min at 14,000 g to recover the supernatant and discard cell debris. The protein concentration was measured by absorbance at 280 nm (A_{280}) using a NanoDrop spectrophotometer.

Samples (20 µg in 50 µL) were loaded onto a 10% SDS-PAGE mini stacking gel (BioRad #4561034) to remove lipids, detergents and salts. The gel band was reduced with 10 mM DTT, alkylated with 55 mM iodoacetic acid and digested with 1 µg trypsin for 18 hrs at 37 °C. Extracted peptides were re-solubilized in 0.1% aqueous formic acid and loaded onto a Acclaim Pepmap pre-column (Thermo, 75 µm ID X 2 cm with 3 µm C18 beads) and then onto an Acclaim Pepmap Easyspray analytical column (Thermo, 75 µM X 15 cm with 2 µM C18 beads) for separation using a Dionex Ultimate 3000 uHPLC at 250 nL/min with a gradient of 2-35% organic solvent (0.1% formic acid in acetonitrile) over 3 hrs. Peptides were analyzed using an Orbitrap Fusion mass spectrometer (Thermo Scientific) operating at 120,000 resolution (Full width at half maximum (FWHM) in MS1) with higher-energy collisional dissociation (HCD) sequencing (15,000 resolution) at top speed for all peptides with a charge of 2+ or greater.

ESKAPE Act Plus pathway activation analysis

Analysis of the complete proteomic datasets was performed using the ESKAPE Act Plus, a Shiny app designed for activation analysis of ESKAPE pathogens and other prokaryotes[1]. Input data included Uniprot protein identifiers and the log2 FC values (ΔSR relative to WT) for all detected proteins. Significant activation or repression was identified using a binomial test (FDR adjusted p-value ≤0.05) accounting for the log2 FC of all terms that fall within a given GO term and tested against the null hypothesis that 50% of the terms have log2 FC>0 and the other 50% have a log2 FC<0.

STRING pathway enrichment and network clustering analysis

We use STRING DB (version 12.0 https://string-db.org/)[2,3] for pathway enrichment and network clustering analysis of differentially abundant proteins (DAP). STRING identifies interactions between proteins using experimental data and computational prediction and identifies pathway enrichment through protein annotation databases such as GO, KEGG and Reactome. The Local Network Cluster enrichment uses precomputed interaction clusters from the full STRING network. The pathways or enrichment terms are ranked based on their Signal, a metric that reflects the weighted harmonic mean of the observed to expected gene ratio and −log(FDR) for an enriched term[2]. To identify functionally related modules within the network, clustering was performed using the Markov Cluster Algorithm (MCL) as implemented in STRING with an inflation parameter of 1.8. The resulting clusters represent putative functional modules that were interpreted based on their protein composition and associated functional annotations.

RNA-seq sample preparation and data analysis

Samples were processed and sequenced commercially (SeqCenter, LLC) as follows. RNA was extracted using the ZymoBIOMICS™ Quick-RNA Miniprep Kit (Zymo Research, R1055). Samples were treated with DNAse (Invitrogen DNAse) and Stranded Total RNA Prep Ligation (Illumina) with Ribo-Zero Plus kit, and 10bp unique dual indices were used for library preparation. Paired-end short-read sequencing was done using the NovaSeq X Plus platform (Illumina), with mean read counts between 24 and 32 million reads for sample. Demultiplexing, quality control, and adapter trimming was performed with Illumina bcl-convert (v4.2.4).

Trimmed reads were mapped to *P. aeruginosa* PAO1 (accession GCF_000006765.1)[4], using STAR (version 2.7.8a)[5]. MultiQC (version 1.11)[6] was used to examine mapping statistics and read quality. Mean read mapping was 10.7 million paired reads per sample (min 8 million, max 15 million). Unmapped reads were checked for contaminants using Kraken (version 2.1.2)[7], using minikraken2 v2 as a database, which showed less than 0.1% contamination.

Whole genome sequencing

Genomic DNA was extracted from PAO1 WT and ΔSR strains using the DNeasy Blood & Tissue Kit (Qiagen #69504) according to the manufacturer's protocols. Whole genome sequencing was

performed using Illumina HiSeq 4000 paired-end short-read sequencing at >200x coverage (Beijing Genome Institute) and assembled using the open-source online platform Galaxy (https://usegalaxy.org/). The reads from ΔSR mutant were aligned to the assembled WT genome and analyzed with the Integrative Genomics Viewer (IGV) to confirm the deletions in the *relA* and *spoT* genes and absence of other mutations.

RT-qPCR

Stationary phase cultures grown as done for the proteomic and RNA-seq analyses and were processed with RNAprotect Bacteria mini kit (Qiagen, #74524). RNA was extracted from 2x10^8 bacteria using the Rneasy Mini kit (Qiagen, #74104) according to the manufacturer's protocol. Total RNA was reverse-transcribed into cDNA using Quantitect Reverse transcriptase kit (Qiagen, #2053110), and amplification was carried out using Power SYBR green PCR master mix (Applied Biosystems, #4367659) with gene specific primers[8–10] (listed in Supp. Table S9) on the Applied Biosystems 7300 instrument.

Target gene expressions were normalized to the geometric mean of two stable reference genes (*proC* and *gyrB*) to generate a robust normalization factor[11,12]. Relative expression levels were calculated using the \(2^{-\Delta \Delta Ct}\) method, with the mean \(\Delta Ct\) of the WT group serving as the calibrator. Statistical significance was determined using an unpaired Student's t-test on the \(\Delta Ct\) values, followed by Benjamini-Hochberg FDR correction for multiple comparisons[13].

PvdA Western blot

Cell lysates were prepared from cultures grown under the same conditions described for proteomic and RNA-seq analyses. Cell pellets from 5 mL overnight cultures were resuspended in 5 mL of lysis buffer (1% Triton X-100, 150 mM NaCl, 1 mM EDTA, 50 mM Tris-Cl (pH 8.0), protease inhibitor cocktail (cOmplete™, Mini Protease Inhibitor Cocktail Ref 04693124001),1 mg/mL lysozyme), incubated on ice for 45 min, followed by sonication (3x 10 sec on/ 30 sec off, 20% amplitude ~100W) on ice, followed by centrifugation at 16 000 x g for 15 min at 4°C to remove debris. Protein concentration of the lysate was confirmed using a Bradford assay (Bio-Rad #5000006).

For Western blot analysis, 30 µg of protein from each sample cell lysate was resolved by SDS-PAGE (10% Acrylamide/Bis, 0.375 M Tris-Cl pH8.8, 0.1% SDS, 0.1% Ammonium Persulfate, and 0.01% TEMED) and transferred onto a nitrocellulose membrane. Following transfer, the membrane was stained with Ponceau S solution to assess protein transfer and loading. The membrane was then blocked with 5% skim milk and 2.5% BSA in Tris-buffered saline containing 0.1% Tween-20 (TBST) for 1 h at room temperature. The membrane was incubated overnight at 4 °C with a 1:2,000 dilution of anti-PvdA primary antibody (Invitrogen, PA5-117666), followed by four 10-min washes with TBST. The membrane was subsequently incubated with 1:15,000 HRP-conjugated secondary antibody (Invitrogen, #31458) for 1 h at room temperature and washed

four times with TBST. Protein bands were visualized using an enhanced chemiluminescence (ECL) detection system and imaged using a digital gel documentation system (Bio-Rad ChemiDoc-MP).

Purified recombinant PvdA (CUSABIO, CSB-EP706553EZX) containing a 6×His tag, was treated with SUMO protease (Sigma-Aldrich, #SAE0067) to remove the tag and 200 ng was used as a positive control. A *pvdA* knockout mutant (PA14 Δ*pvdA* Δ*pchE*[14]) was used as a negative control. PvdA abundance was quantified by band densitometry by normalizing the intensity of the PvdA band (50kD) to the total protein signal in the corresponding lane based on Ponceau S staining using ImageJ software.

Proteomic-mRNA congruency analysis

Identification of incongruent protein-mRNA pairs was performed by aligning the log2 FC (ΔSR - WT) of protein with the corresponding log2 FC of mRNA for each protein. Positive log2 FC indicates upregulation in ΔSR when compared to WT. Incongruence was defined if one metric (either protein or mRNA) was positive and the other was negative. This was determined by multiplying the two values and a positive product (>0) signified congruent expression (both protein and mRNA log2 FC were either positive or negative), while a negative product (<0) indicated incongruent differential expression. To identify pairs with more notable levels of incongruency (filtering out pairs where both protein or mRNA had a near 0 log2 FC) we selected notable incongruent protein-mRNA pairs that were defined by protein and/or mRNA (at least 1) having a |log2 FC|≥ 1. These notable pairs were split into 2 categories: up regulated protein/ down regulated mRNA and down regulated protein/ up regulated mRNA.

CopraRNA

CopraRNA (Freiburg RNA tools) was used to predict the sRNA binding targets of PrrF1 and PrrF2[15–17]. For PrrF1, predictions were pre-computed based on PrrF1 homologues from 7 *Pseudomonas* species and one *Stutzerimonas* species, *Pa* PAO1 (NC_002516), *Pa* DK2 (NC_018080), *P. protegens* Pf-5 (NC_004129), *P. putida* W619 (NC_010501), *P. mendocina* ymp (NC_009439), *P. entomophila* L48 (NC_008027), *P. syringae* pv. tomato str. DC3000 (NC_004578), and *Stutzerimonas stutzeri* RCH2 (NC_019936). For PrrF2, predictions was generated by inputting the PrrF2sequence from 4 *Pseudomonas* species, namely *Pa* PAO1 (NC_002516), *Pa* DK2 (NC_018080), *Pa* AR_0360 (NZ_CP027165), *P. protegens* Pf-5 (NC_004129). All PrrF1 and PrrF2 input sequences for CopraRNA are available in Supp. Table S7.

Quantification of total intracellular glutathione by reverse phase HPLC

Stationary phase cells (~5 x 10^9 CFU) were collected by centrifugation, washed twice with PBS and resuspended in 1M perchloric acid. Samples were vortexed for 1 hr at 25 °C, centrifuged at 12,000 g for 10 min and stored at 4 °C. Total glutathione (GSx), composed of reduced glutathione (GSH) and glutathione disulfide (GSSG), were measured by reverse phase- high performance

liquid chromatography (RP-HPLC) as previously done[18]. Briefly, 50 μL of sample was injected into HPLC and flowed through Ultracarb 5 μm ODS (30) LC column at a rate of 1 mL/min. The post-column eluate was mixed with ortho-phtaldialdehyde (370 μM) in 0.2 M tribasic sodium phosphate, pH 12, and filtered with a 0.45 μm membrane (Waters). The mixture then passed through a 6 m tube submerged in a 70 °C water bath at 1 mL/min. GSx signal was measured by fluorescence (Ex. 336nM/ Em. 420nM) and quantification was determined using standard curves of GSH (1-1000 ng/50 μL) and GSSG (1-100 ng/50 μL).

Pyoverdine production

Bacteria were grown in 200 μL LB Miller (iron replete) or LB with 0.25 mM 2,2'-dipyridyl (iron limiting conditions) in 96 well plates incubated for 18 hrs at 37°C with continuous shaking in a multimode plate reader (BioTek Cytation 5 Agilent, CA). Measurements were taken every 30 min for absorbance at OD_{600} for bacterial density and fluorescence Ex 405 nm / Em 465 for pyoverdine production. Pyoverdine production was reported as RFU normalized to OD_{600}.

Sod activity assay

Stationary phase cells were analyzed for superoxide dismutase activity as previously done[19]. Briefly, ~5 x 10^9 stationary phase cells were pelleted, washed twice and resuspended in 250 μL of PBS. The cell suspension was lysed by sonication on ice (6x 10 sec on/ 45 sec off, 20% amplitude/~100W) and centrifuged for 5 min at 12,000 g to recover the supernatant. Cell lysates were stored at -20 °C until analysis. Sod activity was measured as previously done[19,20]. 5 μL of xanthine oxidase solution (500 μg/mL), 10 μL of cell lysate/ SOD standard between 0.2-2 units/ or 50 mM KPI and 500 μL assay solution (50 mM KPI, 30 μM ferricytochrome C, 100 μM xanthine) were combined. OD_{550} was recorded every 5-10 s for 1 min and the slope was calculated using linear regression. To calculate the percent inhibition the following formula was used: 100x ($slope_{negative}$– $slope_{standard}$ or $slope_{lysate}$)/ $slope_{negative}$. The amount of SOD units in the lysate was calculated using the generated SOD standard curve and was normalized by total protein (Pierce™ BCA Protein Assay Kit, Thermo Fisher Scientific).

Oxidant susceptibility disk diffusion assay

Stationary phase cultures were diluted to 10^6-10^7 CFU/mL and spread on LB agar plate. A 6-mm filter disc (Becton Dickinson) was impregnated with 100 mM paraquat (PQ) or 1.5 μM tert-butyl hydroperoxide (tBHP) and was placed on the agar surface. Plates were incubated at 37 °C for 16 hrs before measuring the zone of inhibition (ZOI) radius. For H_2O_2, 100 μL of stationary phase culture was added to melted 0.6% top agar and poured onto LB agar. 30 % H_2O_2 impregnated disks were placed on the agar overlay and grown for 16 hrs at 37 °C.

References

1. Koeppen, K., Hampton, T. H., Neff, S. L. & Stanton, B. A. ESKAPE Act Plus: Pathway Activation Analysis for Bacterial Pathogens. *mSystems* **7**, e00468-22 (2022).

2. Szklarczyk, D. *et al.* The STRING database in 2025: protein networks with directionality of regulation. *Nucleic Acids Res* **53**, D730–D737 (2025).

3. Szklarczyk, D. *et al.* STRING v11: protein–protein association networks with increased coverage, supporting functional discovery in genome-wide experimental datasets. *Nucleic Acids Research* **47**, D607–D613 (2019).

4. Stover, C. K. *et al.* Complete genome sequence of Pseudomonas aeruginosa PAO1, an opportunistic pathogen. *Nature* **406**, 959–964 (2000).

5. Dobin, A. *et al.* STAR: ultrafast universal RNA-seq aligner. *Bioinformatics* **29**, 15–21 (2013).

6. Ewels, P., Magnusson, M., Lundin, S. & Käller, M. MultiQC: summarize analysis results for multiple tools and samples in a single report. *Bioinformatics* **32**, 3047–3048 (2016).

7. Wood, D. E., Lu, J. & Langmead, B. Improved metagenomic analysis with Kraken 2. *Genome Biol* **20**, 257 (2019).

8. Kim, C., Jothi, R., Oh, K.-K. & Park, D. S. Novel Species-Specific Primers Enable Accurate Detection and Quantification of *Pseudomonas aeruginosa* via qPCR. *Journal of Food Protection* **88**, 100467 (2025).

9. Janssen, K. H. *et al.* Functional Analyses of the RsmY and RsmZ Small Noncoding Regulatory RNAs in Pseudomonas aeruginosa. *J Bacteriol* **200**, e00736-17 (2018).

10. Chourashi, R., Weiner, J. M., Hoang, T.-M., Ouattara, K. & Oglesby, A. G. The Pseudomonas aeruginosa PrrF sRNAs and PqsA promote biofilm formation at body temperature. *J Bacteriol* **208**, e0050725 (2026).

11. Chervoneva, I. *et al.* Selection of optimal reference genes for normalization in quantitative RT-PCR. *BMC Bioinformatics* **11**, 253 (2010).

12. Vandesompele, J. *et al.* Accurate normalization of real-time quantitative RT-PCR data by geometric averaging of multiple internal control genes. *Genome Biol* **3**, RESEARCH0034 (2002).

13. Bustin, S. A. *et al.* The MIQE guidelines: minimum information for publication of quantitative real-time PCR experiments. *Clin Chem* **55**, 611–622 (2009).

14. Wang, Y. *et al.* Phenazine-1-carboxylic acid promotes bacterial biofilm development via ferrous iron acquisition. *J Bacteriol* **193**, 3606–3617 (2011).

15. Wright, P. R. *et al.* CopraRNA and IntaRNA: predicting small RNA targets, networks and interaction domains. *Nucleic Acids Res* **42**, W119-123 (2014).

16. Wright, P. R. *et al.* Comparative genomics boosts target prediction for bacterial small RNAs. *Proc Natl Acad Sci U S A* **110**, E3487-3496 (2013).

17. Raden, M. *et al.* Freiburg RNA tools: a central online resource for RNA-focused research and teaching. *Nucleic Acids Res* **46**, W25–W29 (2018).

18. Ano, S. *et al.* Inflammation and airway hyperresponsiveness after chlorine exposure are prolonged by Nrf2 deficiency in mice. *Free Radic Biol Med* **102**, 1–15 (2017).

19. Martins, D. *et al.* Superoxide dismutase activity confers (p)ppGpp-mediated antibiotic tolerance to stationary-phase *Pseudomonas aeruginosa*. *Proc Natl Acad Sci USA* **115**, 9797–9802 (2018).

20. Forman, H. J. & Fridovich, I. Superoxide dismutase: a comparison of rate constants. *Arch Biochem Biophys* **158**, 396–400 (1973).
